# Spontaneous Cdc42 Polarization Independent of GDI-Mediated Extraction and Actin-Based Trafficking

**DOI:** 10.1371/journal.pbio.1002097

**Published:** 2015-04-02

**Authors:** Felipe O. Bendezú, Vincent Vincenzetti, Dimitrios Vavylonis, Romain Wyss, Horst Vogel, Sophie G. Martin

**Affiliations:** 1 Department of Fundamental Microbiology, University of Lausanne, Lausanne, Switzerland; 2 Department of Physics, Lehigh University, Bethlehem, Pennsylvania, United States of America; 3 Institut des Sciences et Ingénierie Chimiques (ISIC), Ecole Polytechnique Fédérale de Lausanne, Lausanne, Switzerland; Duke University Medical Center, UNITED STATES

## Abstract

The small Rho-family GTPase Cdc42 is critical for cell polarization and polarizes spontaneously in absence of upstream spatial cues. Spontaneous polarization is thought to require dynamic Cdc42 recycling through Guanine nucleotide Dissociation Inhibitor (GDI)-mediated membrane extraction and vesicle trafficking. Here, we describe a functional fluorescent Cdc42 allele in fission yeast, which demonstrates Cdc42 dynamics and polarization independent of these pathways. Furthermore, an engineered Cdc42 allele targeted to the membrane independently of these recycling pathways by an amphipathic helix is viable and polarizes spontaneously to multiple sites in fission and budding yeasts. We show that Cdc42 is highly mobile at the membrane and accumulates at sites of activity, where it displays slower mobility. By contrast, a near-immobile transmembrane domain-containing Cdc42 allele supports viability and polarized activity, but does not accumulate at sites of activity. We propose that Cdc42 activation, enhanced by positive feedback, leads to its local accumulation by capture of fast-diffusing inactive molecules.

## Abstract 2

This study of fission yeast reveals that the active and inactive forms of the small GTPase Cdc42 have different rates of lateral diffusion in the membrane, providing insights into how it becomes spontaneously polarized, thereby determining the polarity of the cell.

## Author Summary

Cell polarization is a critical feature of most cells that underlies their functional organization. A central polarity factor called Cdc42, a small GTPase targeted to the plasma membrane by prenylation, promotes cell polarization in its active GTP-bound form. Cdc42 is a key polarity factor because it accumulates at presumptive sites of polarity, which previous work suggested involves Cdc42 recycling on and off the plasma membrane. In addition, its activity can spontaneously polarize cells in a single location by self-enhancing positive feedback mechanisms, even in the absence of any pre-localized landmarks. In this study, we constructed the first functional fluorescently tagged allele of Cdc42 that replaces the endogenous genomic copy in *Schizosaccharomyces pombe*. This allowed measurements of Cdc42 dynamics at the plasma membrane by live microscopy. Unexpectedly, this approach revealed that Cdc42 primarily moves through lateral diffusion, rather than on and off the plasma membrane. Engineered Cdc42 alleles with alternative membrane-targeting mechanisms demonstrated that Cdc42 activity, indeed, polarizes in the absence of known pathways that recycle Cdc42 on and off the membrane. We further show that the active form, Cdc42-GTP, is less mobile than Cdc42-GDP. We thus propose that Cdc42 polarization occurs as a consequence of its local activation—either through self-enhanced feedback or in response to upstream cues—by a reduction in the active Cdc42 diffusion rate.

## Introduction

Cell polarization is an evolutionary, ancient cellular property that, in eukaryotes, centers around the Rho-family GTPase Cdc42. Cdc42, which cycles between active GTP-bound and inactive GDP-bound forms, is locally activated by Guanine nucleotide Exchange Factors (GEFs) and accumulates at presumptive sites of polarity. Active Cdc42 then promotes the activation of numerous effectors, including p21-activated kinases (PAK), nucleators of actin cytoskeleton assembly, and the exocyst complex for polarized exocytosis [1–3]. Collectively, these pathways transduce the location of Cdc42 activity into effective cell polarization, which underlies essential processes such as proliferation, migration, and signal transduction. Consistent with its central role, the misregulation of Cdc42 and other Rho-GTPases has been implicated in multiple human conditions, such as congenital diseases or infection [4]. Thus, one critical question is, “What are the mechanisms that promote the local activation and accumulation of Cdc42?”

Rho-family GTPases are associated with cellular membranes. The vast majority, including Cdc42, carries a C-terminal CAAX box, which serves as signal for prenylation on the cysteine residue and insertion in the endoplasmic reticulum membrane [5]. From there, the Rho proteins can be distributed through the trafficking system up to the plasma membrane, where Cdc42 localizes. Rho-family GTPases, including Cdc42, can also be extracted from membranes by so-called Guanine nucleotide Dissociation Inhibitors (GDIs), which shield the prenyl group and keep the Rho protein in a soluble cytosolic form [6].

While local activation of Cdc42 may depend on the trivial presence of a pre-localized activator, groundbreaking work in the budding yeast has shown that Cdc42 displays the ability to polarize spontaneously, whereby both its active form and the total protein pool become dynamically polarized, even in absence of pre-established landmarks. Spontaneous polarization—also known as symmetry breaking—is observed in many cell types when the spatial cues normally directing cell polarization, such as external chemo-attractants or internal landmarks, are absent [7,8]. It also occurs naturally, for instance, in germinating yeast spores, which establish polarization in absence of any known pre-localized landmarks [9]. Spontaneous polarization relies primarily on positive feedback mechanisms that amplify initial stochastic noise into robust polarization [7,8]. One such feedback mechanism involves the formation of a protein complex between a Cdc42-GTP-binding effector—a PAK—and a Cdc42 GEF, which propagates Cdc42 activation around clusters of Cdc42 activity [10–13]. However, this autocatalytic self-amplifying system does not by itself explain accumulation of Cdc42 at the site of activity, which is thought to arise from coupling to dynamic recycling of Cdc42.

Two modes of Cdc42 recycling from and to the plasma membrane, with distinct dynamic properties, have been proposed. The first slow mode relies on Cdc42 trafficking on vesicles through endo- and exocytosis [14–18]. As Cdc42 promotes the assembly of actin cables, which serve as tracks for the delivery of secretory vesicles, this, in principle, constitutes a mechanism by which to enrich Cdc42 at sites of Cdc42 activity [19,20]. However, the low concentration of Cdc42 on secretory vesicles has raised debate about whether this feedback would indeed reinforce Cdc42 polarization [18,21,22]. The second fast recycling mode depends on GDI-mediated extraction of Cdc42-GDP [23–25]. As the GDI interaction can be competed out by the GEF, this may also enhance Cdc42 delivery and membrane re-insertion at sites of GEF localization [23,24]. Because simultaneous block of vesicle secretion and GDI deletion leads to loss of GFP-Cdc42 polarization [23,25], the current view is that spontaneous polarization of Cdc42 requires these two recycling routes. However, it is important to note that the N-terminally tagged GFP-Cdc42 fusion used in all dynamic studies to date is not fully functional (see below; [18,23,26,27].

In addition to positive feedbacks, the existence of negative feedback and competition mechanisms has been revealed by the oscillatory behavior of polarity patch components [26,28]. These oscillations were observed in both budding and fission yeasts, with a distinct outcome: whereas these oscillations resolve into a single patch at the prospective bud site in the former, they persist throughout bipolar extension at cell poles in the latter [29]. In fission yeast, bipolar growth occurs after passage through S-phase and requires the Tea1-Tea4 landmark complex, which is deposited at cell poles by microtubules and feeds into the Cdc42 activation cycle [30,31]. The presence of oscillations in both organisms, as well as the existence of mutant conditions allowing the rare formation of two simultaneous buds in *Saccharomyces cerevisiae*, suggests that the transition from one to several polarization sites may be an intrinsic, modular property of the Cdc42 spontaneous polarization system [24,26,32–35], though the specific mechanisms remain unclear.

Here we describe a functional, fluorescently tagged Cdc42 allele in fission yeast, which reveals that Cdc42 is mobile at the plasma membrane independently of GDI and vesicle trafficking. Engineered Cdc42 alleles targeted to the plasma membrane in a prenylation-independent manner demonstrate that Cdc42 spontaneously polarizes, often to multiple sites, independently of these recycling pathways in both fission and budding yeast cells. Our work further reveals that inactive Cdc42 displays fast lateral diffusion and slows down and accumulates as a consequence of its local activation.

## Results

### Construction of Functional Fluorescently Tagged Cdc42 Alleles

**Fig 1:**
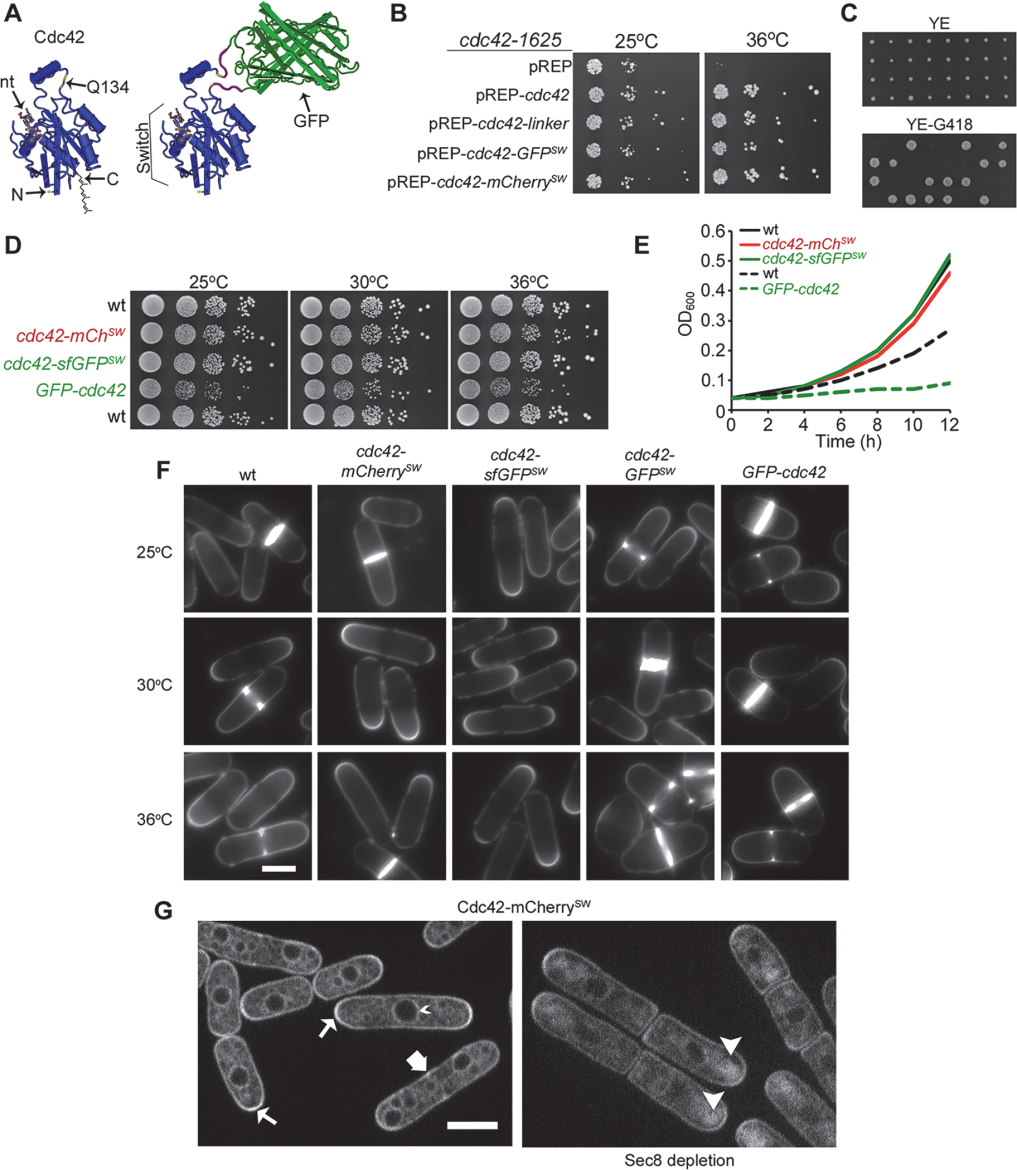
Construction and functional testing of fluorescently tagged *cdc42* sandwich alleles. (A) Structure of Cdc42 (left) and sandwich fusion (right). Note the site of green fluorescent protein (GFP) insertion at Q134 (with spacer sequences in purple) is distant from N and C termini, nucleotide binding pocket (nt) and Switch region. (B) Complementation of *cdc42-1625* temperature sensitivity with pREP41-based plasmids grown on Edinburgh minimal medium (EMM). (C) Tetrad dissection of wt/*cdc42-mCherry* ^*SW*^:*kanMX* diploids on YE (top) and subsequent replica plate on YE G418 (bottom). (D) 10-fold serial dilutions of indicated strains on yeast extract (YE) medium. The top three strains are prototroph, the bottom two strains are double auxotroph for leucine and uracil. (E) Growth curve of cells grown at 30°C in EMM with appropriate supplements. The top three strains are prototroph, the bottom two strains are double auxotroph for leucine and uracil. (F) Calcofluor images of cells grown to log phase at indicated temperatures. (G) Medial spinning disk confocal section of Cdc42-mCherry^SW^ in wild type (wt; left) and following depletion of exocyst component Sec8 (right). Note localization to nuclear membrane (small arrowhead), division site (wide arrow) and enrichment at cell poles (arrows). Arrowheads (right) show sub-apical accumulation of Cdc42-mCherry^SW^. Bars = 5 μm.

To better understand the mechanisms underlying Cdc42 dynamics in live cells, we set out to construct a functional fluorescent fusion protein in fission yeast. Existing fusions in various organisms fuse fluorescent proteins to Cdc42 N-terminus, as the C-terminus is subject to post-translational modification by prenylation (Fig. 1A). However, when this has been tested by gene replacement, these N-terminal fusions compromise Cdc42’s functions, yielding temperature-sensitivity and failure to associate with post-Golgi vesicles in *S*. *cerevisiae* [18,23,26] or altered cell morphology in *Schizosaccharomyces pombe* [27]. Indeed, the *GFP-cdc42* strain displayed slow growth and aberrant morphology at all tested temperatures (see Fig. 1D–F). We chose the alternate approach of placing a fluorescent protein gene within the *cdc42* reading frame, a strategy previously tried with success on other proteins [36,37].

Potentially permissive sites for fluorophore insertion were determined by examining Cdc42 crystal structure and looking for solvent-exposed poorly-conserved external loops distant from the switch regions and α2 helix that mediate the interface of most known interactors (Fig. 1A) [38]. The α3′ helix fit these criteria well and the linker-SGGSACSGPPG- was inserted following amino acid Q134. Expression of Cdc42-Q134-linker from a plasmid complemented the *cdc42-1625* temperature sensitive mutant at restrictive temperature (Fig. 1B). Insertion of either GFP or mCherry at this site, generating sandwich fusions Cdc42-GFP^SW^ or Cdc42-mCherry^SW^, likewise complemented the *cdc42-1625* mutant (Fig. 1B). We next engineered strains expressing the sandwich fusions as the sole source of Cdc42 from its native genomic promoter. Replacement of *cdc42* in diploid cells followed by germination of haploid spores yielded colonies of equal size (Fig. 1C). Proper integration in the genome was confirmed by diagnostic PCR and Southern blotting (S1A–C Fig). Remarkably, cells expressing Cdc42-mCherry^SW^ or Cdc42-sfGFP^SW^ (superfolder-GFP) showed growth rate, cell width and length at division, and division plane positioning indistinguishable from wild type, even at temperatures up to 36°C (Fig. 1D–F, S1D Fig). We note that Cdc42-GFP^SW^ was deficient at elevated temperatures (Fig. 1F), suggesting that the slow rate of GFP folding slightly impairs the functionality of the fusion protein [39,40]. We thus used Cdc42-mCherry^SW^ or Cdc42-sfGFP^SW^ in all subsequent experiments.

Cdc42-mCherry^SW^ was enriched at the cell tips and division sites (Fig. 1G). Significant levels of Cdc42-mCherry^SW^ were also found along the cell sides and on internal membranes including the nuclear and presumably vacuolar membranes. Cdc42-sfGFP^SW^ showed similar localization (S1E Fig). In cells depleted of the exocyst member Sec8, in which exocytic vesicles accumulate but fail to fuse at the cell tip [41,42], Cdc42-mCherry^SW^ accumulated sub-apically, confirming that Cdc42 is trafficked on exocytic vesicles (Fig. 1G). Finally, we found no synthetic interactions with any of the mutants used below. Thus, at least during mitotic growth, the mCherry and sfGFP sandwich constructs appear to be functional fusions to Cdc42.

### Enrichment of Cdc42-GTP at Sites of Polarity

**Fig 2:**
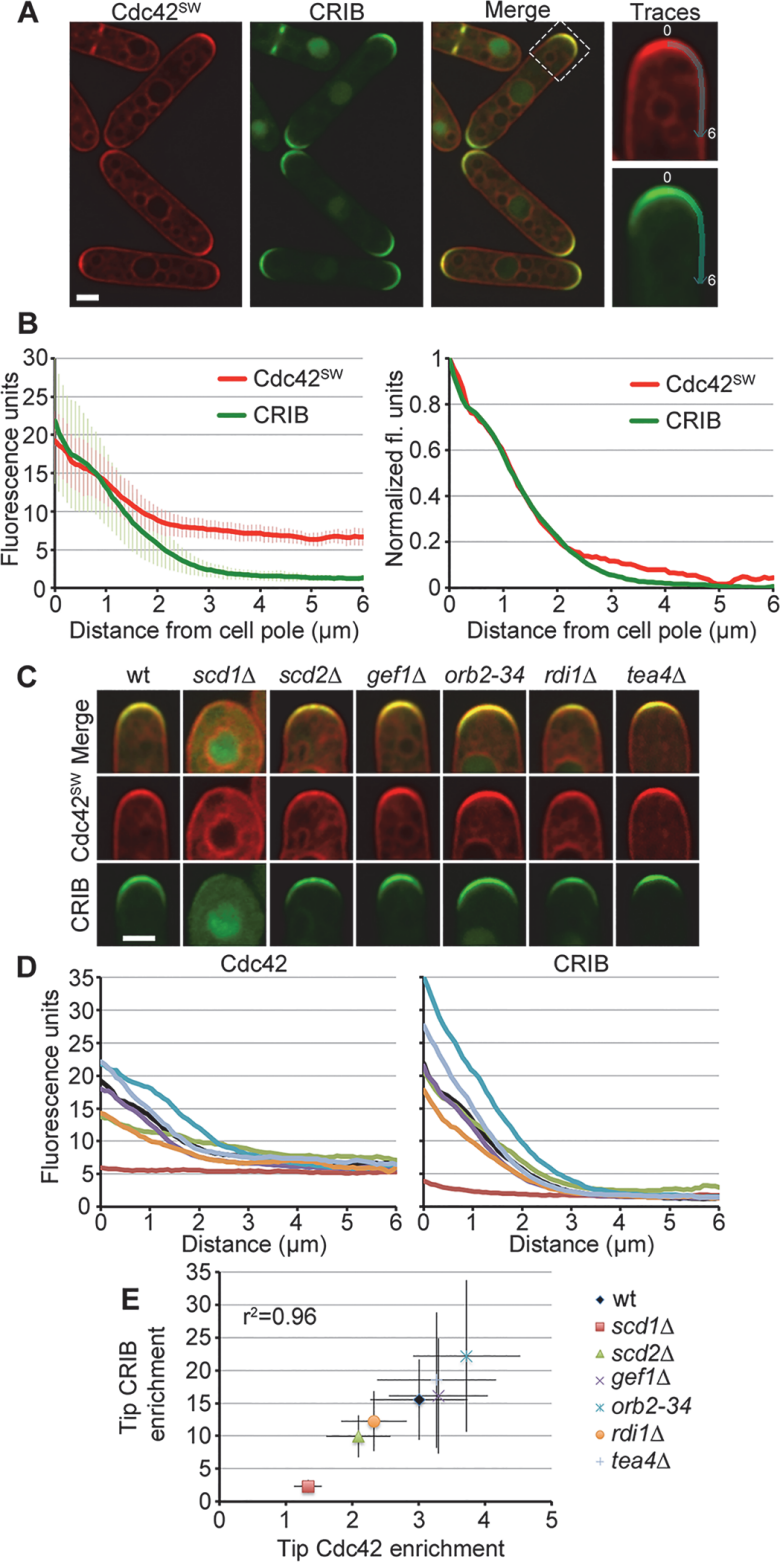
Enrichment of GTP-Cdc42 at sites of polarity. (A) Cdc42-mCherry^SW^ and CRIB-3GFP localization. Traces for cortical measurements are shown on the right. (B) Average profiles of fluorescence intensity along cortical traces with standard deviation (left) and after normalization to the maximum and minimum intensity values (right). (C) Representative Cdc42-mCherry^SW^ and CRIB-3GFP images at cell poles in wt and indicated mutant cells. (D) Average profiles of cells shown in C. (E) Plot of Cdc42 versus CRIB fluorescence at the tip showing that the enrichment of total Cdc42 correlates with its activity. *n* ≥ 40 for each profile or data point. Bars = 2 μm.

To quantitatively define the distribution and activity of Cdc42 at the plasma membrane, we co-imaged Cdc42-mCherry^SW^ with CRIB-3GFP, a probe that selectively binds active Cdc42-GTP (CRIB stands for Cdc42- and Rac-interactive binding domain) [43]. The distribution of CRIB closely mirrored that of Cdc42 enrichment at cell poles (Fig. 2A). At cell poles with strong CRIB localization, Cdc42 was enriched 3-fold (± 0.7, *n* = 40) over its levels at cell side (Fig. 2B). At cell poles with low CRIB levels, these correlated with low Cdc42 enrichment (S2 Fig). Normalization of the Cdc42 and CRIB distribution profiles to their maximum and minimum yielded overlapping curves with identical decay rates, suggesting a very tight correlation between enrichment of Cdc42 and the active form (Fig. 2B right).

Examination of Cdc42-mCherry^SW^ and CRIB-3GFP in a panel of mutants shown or predicted to regulate Cdc42 activity further strengthened this correlation (Fig. 2C). Indeed, Cdc42 tip enrichment strongly correlated with CRIB tip enrichment across all mutants examined (linear regression r^2^ = 0.96; Fig. 2D–E). Two mutants—*orb2-34*, a largely inactive allele of the PAK kinase Shk1/Pak1 proposed to act in a negative feedback to inhibit Cdc42 activity [28], and deletion of the Tea4 landmark [44,45]—showed higher average Cdc42 activity and enrichment at their single growing cell tip. Deletion of the Cdc42 GEF Scd1 [46] showed dramatic loss of Cdc42 local activity and enrichment. By contrast, Cdc42 activity and enrichment were not as severely affected by deletion of the putative scaffold Scd2, which forms a complex with Scd1 and Cdc42 [46]. Finally, deletion of the second Cdc42 GEF Gef1 [47,48], or of the only predicted GDI Rdi1 [49], had no or minor effect on Cdc42 local activity and enrichment. In summary, these data indicate that the accumulation of Cdc42 at growing cell poles represents the active form.

### Distinct Mobility of Cdc42 at Cell Tips and Cell Sides

**Fig 3:**
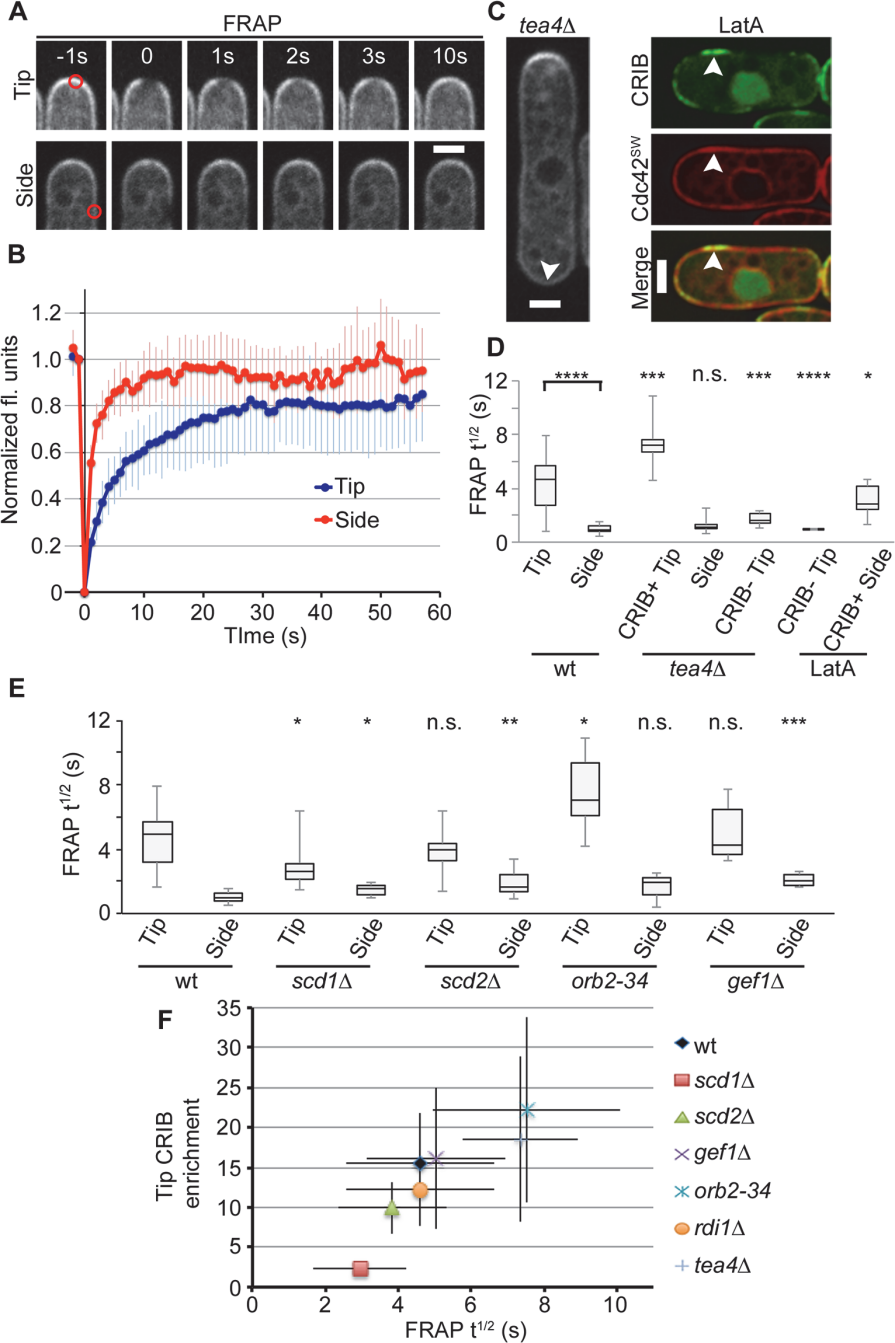
Correlation between Cdc42 dynamics and activity. (A) Example time series FRAP experiment following Cdc42-mCherry^SW^ bleach at cell tip and side. Bleach area is indicated in red. (B) Average recovery of Cdc42-mCherry^SW^ at cell tip and side (standard deviation shown, *n* = 12). (C) Control images for influence of pole geometry on FRAP recovery. *tea4*Δ cells grow in a mono-polar manner. Arrowhead indicates non-growing end. Prolonged treatment with LatA leads to cell side activation and accumulation of Cdc42 (arrowhead). (D) Halftimes (t^1/2^) of Cdc42-mCherry^SW^ FRAP recovery for indicated conditions. CRIB+ designates CRIB presence; CRIB-absence of CRIB signal. Asterisks indicate statistically significant differences between cell tip and cell side for wild type, between mutant or drug-treated tip versus wild-type tip, and between mutant or drug-treated side versus wild-type side. (E) Halftimes of Cdc42-mCherry^SW^ FRAP recovery for indicated strains. Asterisks indicate statistically significant differences between mutant tip or side versus wild-type tip or side, respectively. (F) FRAP halftimes correlate with activity levels of Cdc42. Standard deviation is shown. *n* ≥ 12 for each profile or data point. Bars = 2 μm. Student’s *t* tests were used, with the following notation: n.s. = *p* > 0.05 * is *p* ≤ 0.05, ** is *p* ≤ 0.01, *** is *p* ≤ 0.001, **** is *p* ≤ 0.0001.

We used fluorescence recovery after photobleaching (FRAP) experiments to measure the mobility of Cdc42 at the plasma membrane. Using an identical 0.9 μm bleach spot in all experiments, we found that Cdc42-mCherry^SW^ fluorescence recovers significantly faster at cell sides than cell poles, with recovery halftimes of 1.0 ± 0.3 versus 4.6 ± 2.0 s^−1^, respectively (Fig. 3A,B). Thus, Cdc42 is highly mobile at the plasma membrane, but significantly slower at cell tips (Student’s *t* test, *p* = 3.5 x 10^−5^).

We considered whether cell geometry may cause this difference by examining Cdc42 mobility at cell tips lacking Cdc42 activity or at sites of activity on cell sides. Cdc42 mobility at the non-growing cell tip of *tea4*Δ cells, which has vastly reduced Cdc42 activity and enrichment (Fig. 3C,D), was significantly higher than at the other cell tip and more similar to the cell sides. Conversely, we generated zones of Cdc42 activity and enrichment at cell sides by treating cells with the actin depolymerizing drug Latrunculin A (LatA) for 30–40 min (Fig. 3C,D, see below). This leads to progressive loss of CRIB from cell poles and formation of dynamic zones of CRIB on cell sides [42]. Cdc42 was enriched in these zones and displayed significantly slower mobility, whereas Cdc42 from depleted cell poles showed fast mobility. Thus, the geometry of the cell tip does not constrain Cdc42 mobility, and Cdc42 mobility can be slowed down also at cell sides.

Measurement of Cdc42 FRAP halftimes in the panel of regulator mutants described above further established a correlation between the levels of active Cdc42, as detected by CRIB, and Cdc42 slow mobility (high FRAP halftimes) at cell tips (Fig. 3E,F; linear regression r^2^ = 0.82). Cdc42 mobility was high (low FRAP halftimes) at the sides of all mutants, though some differences were noticed in comparison to wild type. We also tested the mobility of the Cdc42 GTP-locked allele, Cdc42^Q61L^ *-mCherry* ^*SW*^ expressed from plasmids under control of an inducible promoter in a *cdc42-sfGFP* ^*SW*^ strain. Long-term induction led to cell rounding, as previously reported for untagged Cdc42^Q61L^ (S3A Fig) [50]. FRAP experiments performed after short-term induction before cell shape change showed slow mobility of Cdc42^Q61L^ *-mCherry* ^*SW*^ at both cell tips and cell sides, with halftimes similar or higher than those of wild-type Cdc42 at cell tips (S3B,C Fig). We note that Cdc42^Q61L^ *-mCherry* ^*SW*^ expression had no effect on the dynamics of Cdc42-sfGFP^SW^ on cell sides, but led to reduction in its halftime at cell tips, suggesting titration of some factor for Cdc42 activation or stabilization. Cdc42 dynamics remained slow at cell tips and fast at cell sides in both channels when wild-type Cdc42*-mCherry* ^*SW*^ was co-expressed from plasmids in a *cdc42-sfGFP* ^*SW*^ strain. We conclude that Cdc42-GTP exhibits slower mobility than Cdc42-GDP at the plasma membrane.

### Cdc42 Mobility Is Largely Independent of GDI or Vesicle Trafficking

**Fig 4:**
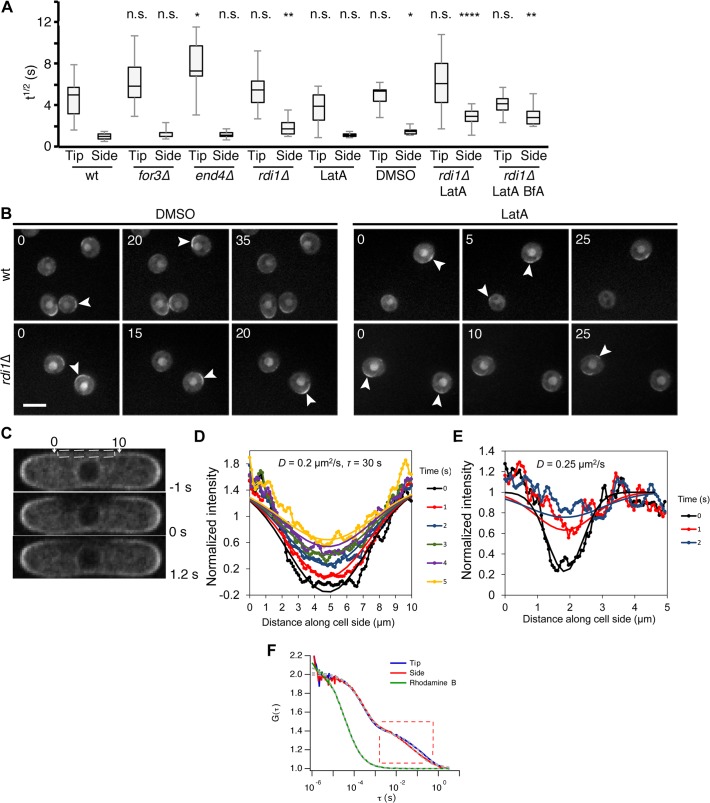
Cdc42 dynamics at the plasma membrane are largely independent of GDI or vesicle trafficking and strongly diffusive. (A) FRAP halftimes (t^1/2^) of Cdc42-mCherry^SW^ recovery for indicated mutants and drug treatments. *n* ≥ 12 for wt, *rdi1*Δ, and *rdi1*Δ LatA. *n* ≥ 7 for all others. The asterisks indicate statistically significant differences between mutant tip or side versus wild-type tip or side, respectively, in a Student’s *t* test in which n.s. = *p* > 0.05, * is *p* ≤ 0.05, ** is *p* ≤ 0.01, *** is *p* ≤ 0.001, and **** is *p* ≤ 0.0001. (B) CRIB-3GFP in wt and *rdi1*Δ spores on rich YE media with either DMSO or LatA. Arrowheads indicate zones of active Cdc42. Time is shown in minutes. Scale bar = 5μm. (C) Cdc42-mCherry^SW^ images at indicated time points relative to large cortical side bleach (dashed box). (D) Intensity profile along cell side versus time for cell in panel C. The intensity along the membrane was measured by fitting an active contour to the cell boundary and integrating the intensity within 3 pixels. Continuous lines show fit to a model of recovery with diffusion coefficient *D* and uniform cytoplasmic exchange with time constant *τ* (see Materials and Methods). (E) Same as panel D but for a smaller bleached region (0.9 μm) exhibiting faster recovery. This difference indicates that the recovery of the smaller bleached region is dominated by diffusion. (F) Normalized fluorescence correlation spectroscopy (FCS) autocorrelation curves of calibration dye Rhodamine B (green) and of Cdc42-mCherry^SW^ at side (red) and tip locations (blue). The curve showing a slow diffusion component corresponds to the membrane-inserted prenylated species (see S5G Fig). Data were collected from a pool of eight cells and the curves were fitted with a two-component diffusion model (grey dashed lines).

We were surprised to discover that deletion of *rdi1* did not affect Cdc42 mobility at cell tips in the experiment above (Fig. 3F, Fig. 4A). Though Rdi1 is the sole predicted GDI in *S*. *pombe*, its deletion yields only a minor morphological phenotype, with cells slightly shorter and wider than wild-type cells at division (S4A–D Fig). Disruption of actin cables in formin *for3*Δ mutant [51], interference with endocytosis in *end4*Δ mutant [52], or disruption of all actin structures by treatment with 200 μM LatA for a short time (5–10 min), also had no or minor effect on Cdc42 mobility (Fig. 4A). Remarkably, Cdc42 mobility at cell tips was even maintained in *rdi1*Δ mutant cells treated with LatA. Further collapse of the membrane trafficking system by treatment with Brefeldin A (BFA) also failed to slow down Cdc42 dynamics at cell tips (Fig. 4A). We note, however, that *rdi1* deletion slightly slowed down Cdc42 mobility at cell sides especially in combination with actin cytoskeleton disruption, though the absence of known actin structures or trafficking pathways at cell sides suggests the effect of actin disruption may be indirect.

These data indicate that Cdc42 dynamics at the plasma membrane occurs largely independently of GDI-mediated membrane extraction and vesicle trafficking. Furthermore, long-term (30–40 min) LatA treatment led to the formation of new zones of active Cdc42 polarization at cell sides, which were dynamic, forming and disappearing over time, even in *rdi1*Δ cells (S4E Fig). Finally, zones of active Cdc42 formed spontaneously in spores [9], even upon removal of both GDI and actin structures (Fig. 4B). Thus, Cdc42 mobility and its ability to locally accumulate require neither GDI nor actin-dependent vesicle trafficking, though the actin cytoskeleton is required for maintenance of an active Cdc42 zone at a stable location.

### Cdc42 Diffuses Laterally at the Plasma Membrane

The FRAP measurements described above may reflect exchange of Cdc42 between the membrane and the cytosol or lateral diffusion along the membrane. In case of lateral diffusion, the rate of fluorescence recovery decreases with increasing size of the bleach zone. Photobleaching of Cdc42 over wider (4–6 μm) zones at the sides of wild-type, *rdi1*Δ, and *rdi1*Δ cells treated with LatA yielded significantly slower recovery, suggesting significant contribution of lateral diffusion to Cdc42 dynamics (Fig. 4C–E).

The recovery on the cell sides was fitted with a model that accounts for 2-D membrane diffusion and uniform exchange with a fast-diffusing cytoplasmic pool (Fig. 4D,E). Cytoplasmic exchange results in exponential recovery over time while membrane diffusion results in algebraic recovery of the intensity at the center of the bleached and marginally detectable broadening of the bleached region over time. The model predicts the evolution of the initial Gaussian-shaped bleach profile as a function of distance along the cell contour and time, with the diffusion coefficient and exchange time as fitting parameters. Fits to the diffusion-dominated recovery of narrow bleached regions give a diffusion coefficient ranging between 0.15 and 0.35 μm^2^/s in wild-type and *rdi1Δ* cells (Fig. 4E). Use of this range of diffusion coefficient values provides good fits to the recovery of large bleached zones, with a cytoplasmic exchange time longer than 20 s in wild-type and *rdi1Δ* cells (Fig. 4D and S5F Fig). These values for the exchange time suggest that membrane removal, with or without GDI, is a small contribution to kinetics over the diffusion time across the cell side. In *rdi1*Δ cells treated with LatA, diffusion coefficients of 0.1–0.2 μm^2^/s and no exchange component provided good fits. We conclude that lateral diffusion of inactive Cdc42 at cell sides is a major component of Cdc42 mobility.

Photobleaching of half-cell tips also showed recovery from the sides of the bleached zone, with concomitant fluorescence loss in the adjacent tip region, indicating lateral movement along the tip membrane at a slower rate compared to cell sides (S5A–E Fig). Diffusion at cell tips may reflect spatially dependent inter-conversion between fast diffusing Cdc42-GDP and less mobile Cdc42-GTP, which complicates a precise calculation of the Cdc42-GTP diffusion coefficient. However, measurements of the rate of bleached region broadening, as well as fits to a model of diffusion-dominated recovery over a small bleached tip area, suggest at least a 10-fold smaller lateral diffusion coefficient for Cdc42-GTP.

We used fluorescence correlation spectroscopy (FCS) to investigate the mobility properties of Cdc42 further. At the cell sides, the FCS autocorrelation function showed two components with distinct diffusion regimes (Fig. 4F, S5G Fig). The sub-ms component is attributed to cytosolic diffusion, while the slower component depends on the presence of prenylation and hence is associated to a membrane-bound species. A fit with a two-component model revealed a diffusion rate of 0.18 μm^2^/s for the membrane-associated species, in agreement with the FRAP fit above. At the cell tips, FCS did not detect a slower diffusing species, instead only revealing diffusion similar to that observed on cell sides (Fig. 4F and S5G–I Fig). Our inability to detect a slower diffusing components stems from the fact that in standard FCS, to obtain statistical relevance the measuring time has to be 10^3^–10^4^-fold longer than the diffusion time to be determined [53]. This would require a recording time of 15–150 min with an accuracy of 100 nm, incompatible with cell and optical focus stability. As FRAP measures a large ensemble of fluorophores over a much larger area, it can detect much slower diffusing components, but without resolving multiple diffusing components, especially if one (e.g., the slow diffusing component) is dominating. Therefore, by resolving the faster component, our FCS measurements are complementary to the FRAP experiments and suggest that Cdc42-GDP is able to diffuse into the cell tip region. In summary, Cdc42-GDP diffuses at rates of about 0.2 μm^2^/s, whereas Cdc42-GTP diffuses at least 10-fold slower.

### Cdc42 Enrichment Is Not Necessary for Polarized Activity

**Fig 5:**
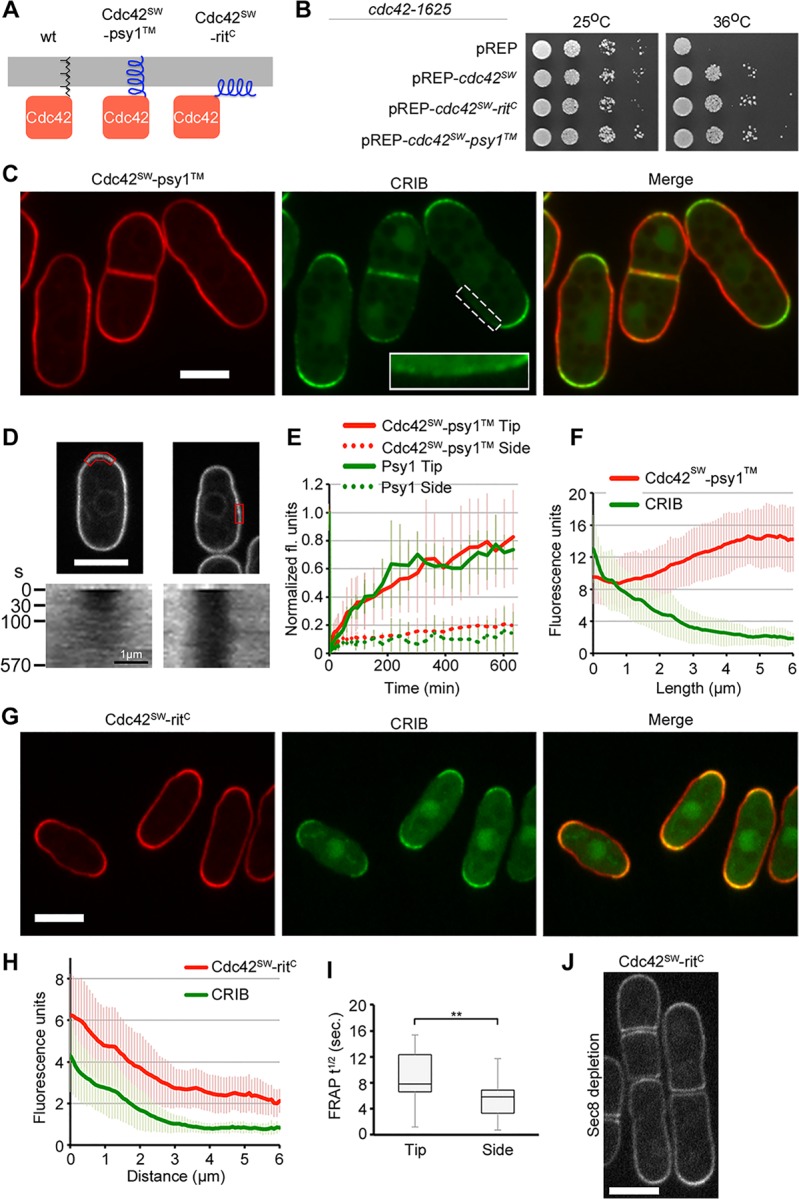
Cdc42 alleles with altered plasma membrane targeting support viability and polarized growth. (A) Schematic of constructs replacing the Cdc42 prenylation domain (left) with the transmembrane domain of tSNARE Psy1 (middle; psy1^TM^) or the amphipathic helix from Rit (right; rit^C^). All constructs are mCherry sandwich fusions. (B) Complementation of *cdc42-1625* temperature sensitivity by pREP41-based plasmids.(C) Cdc42-mCherry^SW^-psy1^TM^ and CRIB-3GFP localization in strain expressing this allele as sole *cdc42* copy. (D) Kymographs (bottom) showing Cdc42-mCherry^SW^-psy1^TM^ recovery in bleached region (red box, top). (E) Average FRAP recovery of Cdc42-mCherry^SW^-psy1^TM^ and tSNARE GFP-Psy1 at cell tips and sides. *n* = 6. (F) Average profiles of fluorescence intensity along cortical traces with standard deviation. Note activity of Cdc42 at the cell pole does not correlate with accumulation. *n* = 40. (G) Cdc42-mCherry^SW^-rit^C^ and CRIB-3GFP localization in strain expressing this allele as sole *cdc42* copy.

To strengthen our findings that GDI and vesicle trafficking only play a minor role on Cdc42 dynamics and further test the role of Cdc42 membrane attachment in cell polarization, we engineered Cdc42 alleles with alternative plasma membrane targeting mechanisms (Fig. 5A). Membrane targeting is essential to Cdc42 function as shown by the fact that removal of the CAAX sequence yielded a diffuse, non-functional Cdc42 allele unable to complement the *cdc42-1625* temperature-sensitive mutant (S6A,B Fig). Remarkably, two distinct Cdc42 alleles in which the CAAX sequence is replaced by a trans-membrane sequence (*cdc42-psy1* ^*TM*^), or an amphipathic helix (*cdc42-rit* ^*C*^), were able to complement the *cdc42-1625* mutant when expressed from plasmids (Fig. 5B) and were viable when integrated as single *cdc42* copy at the native *cdc42* genomic locus. All experiments presented below use strains with these alleles as single *cdc42* copy.

(H) Average profiles of fluorescence intensity along cortical traces with standard deviation. *n* = 40. (I) Halftimes of Cdc42-mCherry^SW^-rit^C^ FRAP recovery at cell tips and sides. *n* ≥ 18. The two values are statistically significantly different (Student’s *t* test *p*-value = 0.0056). (J) Cdc42-mCherry^SW^-rit^C^ localization following depletion of exocyst component Sec8. Note that Cdc42-mCherry^SW^-rit^C^ fusion does not accumulate sub-apically as wild type does (see Fig. 1G, right). Bars = 5 μm.

Cdc42-psy1^TM^, containing the trans-membrane domain of the t-SNARE syntaxin-like protein Psy1 [54], localized to the plasma membrane, where it was almost immobile at cell sides and displayed slow turnover at cell tips (FRAP halftime > 1.5 min), similar to endogenous Psy1 (Fig. 5D,E). This allele did not accumulate at cell poles, where it was less abundant than along cell sides (Fig. 5F). Remarkably, however, these cells localized CRIB-3GFP and polarized growth to both cell poles (Fig. 5C), indicating Cdc42-psy1^TM^ is active at cell poles. We hypothesize that the membrane insertion of Cdc42-psy1^TM^ prevents its rapid recycling and accumulation at sites of activity. We conclude that accumulation of Cdc42 is not absolutely necessary for, and occurs as a consequence of, its local activation.

Though viable, Cdc42-psy1^TM^ cells displayed irregular shapes with variable cell width, with low amounts of CRIB-3GFP also detected on cell sides (Fig. 5C inset; S6C Fig). We tested whether this allele supports the formation of new sites of polarization upon long-term LatA treatment. This led to increased occurrence of CRIB zones on cell sides, similar to our observations in wild-type cells, though the zones were often less well defined, and Cdc42-psy1^TM^ was not enriched in these zones (S6D Fig). While this result shows that Cdc42-psy1^TM^ can support spontaneous polarization to some extent, it also suggests that this almost immobile Cdc42 allele is compromised in its ability to form a focal, well-defined growth zone. We were unable to examine whether this allele can support spontaneous polarization in spores, because of difficulty in obtaining homozygous *cdc42-psy1* ^*TM*^ mutant zygotes. The Cdc42-psy1^TM^ allele also showed synthetic defects with deletion of the landmark Tea1 (S6F Fig). Tea1, which marks cell poles for growth, is required for bipolarity as well as for the maintenance of the rod shape, as *tea1* deletion produces curved, occasionally T-shaped, monopolar cells [55]. Double *cdc42-psy1* ^*TM*^ *tea1*Δ mutants were slow-growing and displayed very aberrant shapes, suggesting that Cdc42-psy1^TM^ activation at cell tips largely relies on upstream polarization cues.

### Symmetry-Breaking by a GDI-Insensitive Cdc42 Allele Not Localized to Exocytic Vesicles

The Cdc42-rit^C^ fusion contains the C-terminal amphipathic helix of a heterologous mammalian protein Rit, previously shown to efficiently localize to the plasma membrane but not to endomembrane systems [56,57]. Cdc42-rit^C^ localized specifically to the plasma membrane and was not detected on endomembranes (Fig. 5G). It also did not accumulate sub-apically in cells depleted of the exocyst member Sec8, indicating that this allele is not trafficked on exocytic vesicles (Fig. 5J). This allele is also predicted not to be a substrate for GDI, because the prenyl group is required to bind GDI [49,58,59]. Remarkably, cdc42-rit^C^ mutant cells grew at near wild-type rates at 25°C and 30°C, though it was compromised at high temperatures, and showed only minor morphological defects, with slightly wider and shorter cells than wild type (S6C,G Fig). Cdc42-rit^C^ accumulated at cell poles, where it was active and enriched 3.1-fold (±1.0, *n* = 40) over its levels at cell sides, similar to wild-type Cdc42 (Fig. 5H). It also showed slower FRAP recovery at cell poles compared to cell sides, though the FRAP halftime at cell sides was about 4-fold slower than wild type (Fig. 5I). The minor phenotype displayed by *cdc42-rit* ^*C*^ mutant cells is consistent with the minor role played by GDI and vesicle trafficking in Cdc42 dynamics.

**Fig 6:**
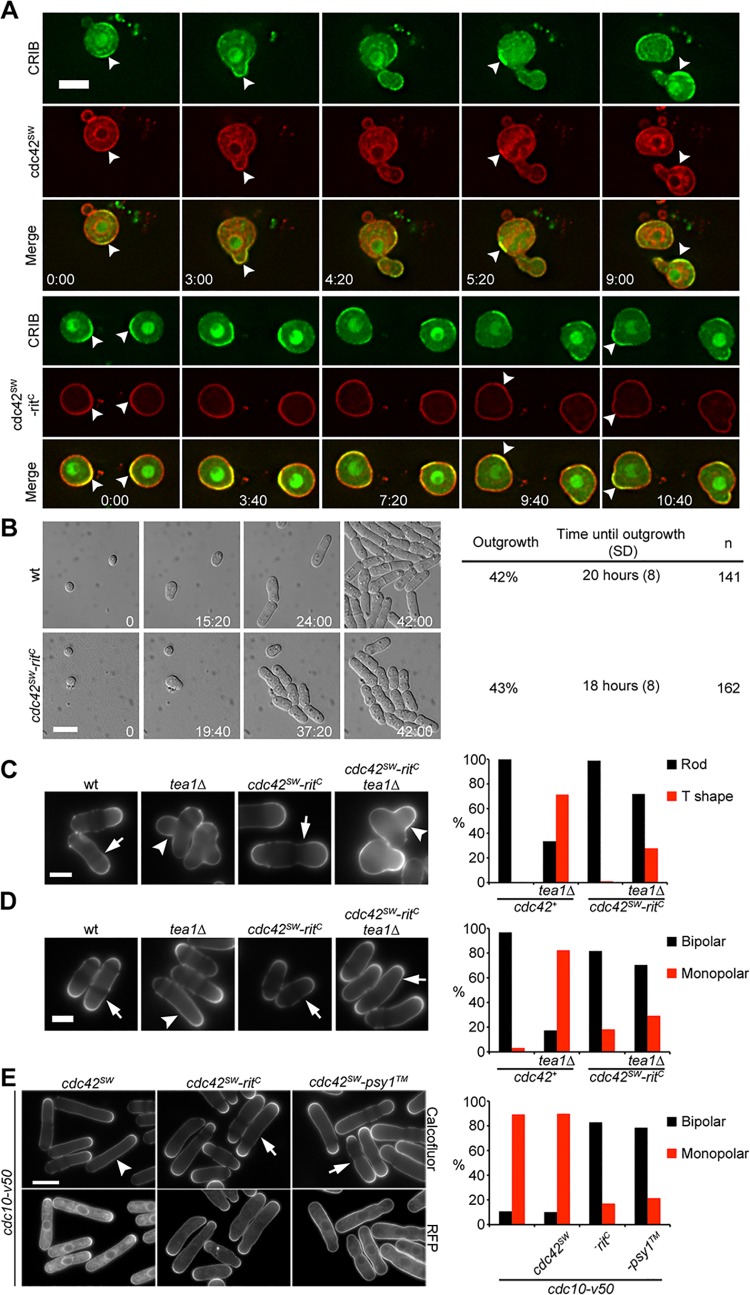
Cdc42-mCherry^SW^-rit^C^ polarizes spontaneously and promotes bipolar growth. (A) CRIB-3GFP and Cdc42-mCherry^SW^ (top) or Cdc42-mCherry^SW^-rit^C^ (bottom) in recovering protoplasts (cell wall digested). Arrowheads show zones of CRIB-labeled active Cdc42. Time is indicated in hours. (B) Differential interference contrast (DIC) images (right) and quantification (left) of wt and *cdc42-mCherry* ^*SW*^ *-rit* ^*C*^ spore outgrowth on rich medium. Time is indicated in hours. (C) Calcofluor images of indicated strains 4 h after refeeding from starvation. Graph shows percent of cells that establish new growth at existing cell poles (rod, arrow) or at novel site at cell middle (T shape, arrowhead). (D) Calcofluor images of indicated strains in log phase. Graph shows percent of cells growing in monopolar (arrowhead) or bipolar (arrow) manner. (E) Calcofluor and mCherry fluorescence images of *cdc10-v50* mutants with indicated *cdc42* alleles blocked in G1 at 36°C. Graph shows percent cells growing in a monopolar (arrowhead) or bipolar (arrow) manner. Bars = 5 μm.

We used four distinct assays to test whether Cdc42-rit^C^ was able to break symmetry in absence of upstream polarity cues. First, upon long-term LatA treatment, Cdc42-rit^C^, like wild-type Cdc42, enriched to novel active zones on cell sides, which are likely devoid of landmarks (S6E Fig). Second, after cell wall digestion generating round protoplasts, both wild-type Cdc42 and Cdc42-rit^C^ enriched in dynamic peripheral active zones during protoplast recovery (Fig. 6A). Third, *cdc42-rit* ^*C*^ mutant spores germinated and polarized growth as efficiently as wild-type spores (Fig. 6B and S7A). Finally, in absence of the landmark Tea1, *cdc42-rit* ^*C*^ mutants formed T-shapes upon re-feeding, indicating polarization at cell sides in absence of the landmark (Fig. 6C). Unexpectedly, and in contrast to *tea1*Δ single mutants, *cdc42-rit* ^*C*^ *tea1*Δ double mutants grew in a bipolar manner in exponential phase (Fig. 6D). This finding may explain the reduced efficiency in the formation of T-shapes, because of competition with growing cell poles [60]. We conclude that Cdc42-rit^C^ is able to break symmetry in absence of upstream cues. Together with our dissection of Cdc42 dynamics, these data show that spontaneous polarization of Cdc42 activity and localization does not require GDI-mediated extraction and actin-based vesicle trafficking.

### Cdc42 Alleles with Altered Membrane Targeting Promote Bipolarity

We were intrigued by the observation that Cdc42-rit^C^ confers bi-polarity in absence of Tea1. In addition to the role of the Tea1/Tea4 landmark, bipolar growth normally is controlled by the cell cycle and occurs only after passage through S-phase, such that *cdc10-v50* mutant cells blocked in G1 phase remain monopolar [31,61]. Remarkably, both Cdc42-rit^C^ and Cdc42-psy1^TM^ promoted bipolar growth in *cdc10-v50* G1-arrested cells (Fig. 6E). Cdc42-rit^C^ and Cdc42-psy1^TM^ mutant cells also displayed clear bipolarity when examined in time-lapse imaging (S7B Fig). Thus, both Cdc42 alleles with altered plasma membrane targeting override the normal regulation to promote bipolar polarization and growth.

### Cdc42-rit^C^ Supports Symmetry Breaking and Causes Multi-bud Formation in *S*. *cerevisiae*

**Fig 7:**
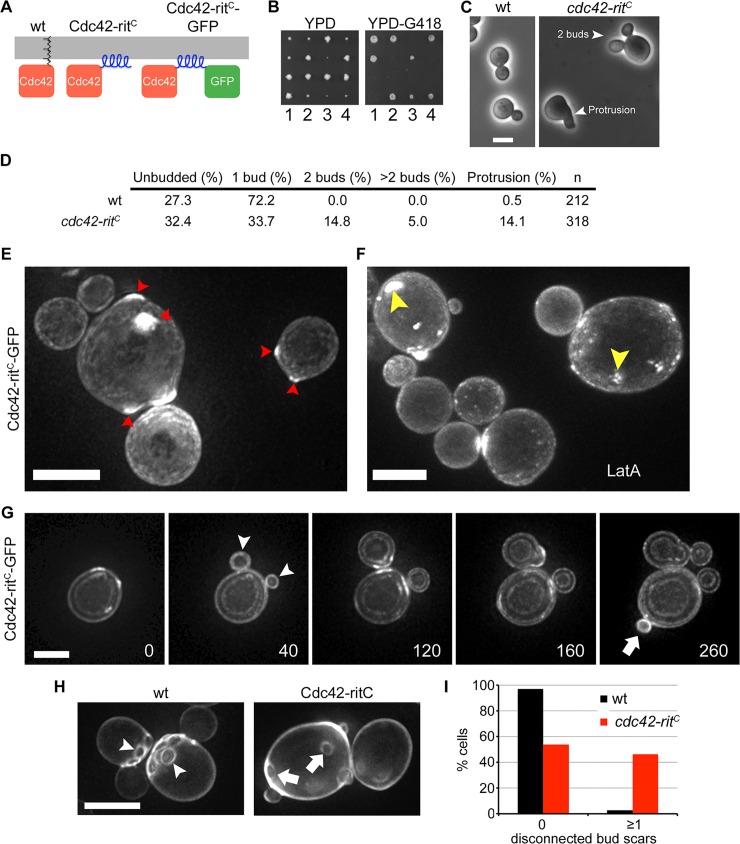
Cdc42-rit^C^ supports viability and polarizes spontaneously in *S*. *cerevisiae*. (A) Schematic of constructs replacing the *S*. *cerevisiae* Cdc42 prenylation domain with the amphipathic helix from Rit (rit^C^). Note GFP coding sequence follows the amphipathic helix. (B) Tetrad dissections of wt/*cdc42-rit* ^*C*^ *-kanMX* diploids on rich YPD media and resulting replica plate onto YPD-G418. Note *cdc42-rit* ^*C*^ *-kanMX* haploids are impaired for growth. (C) Phase images of wt and *cdc42-rit* ^*C*^ cells in log phase. (D) Percent of cells in log phase with indicated phenotypes. (E,F) Maximum projection images of Cdc42-rit^C^-GFP fluorescence without (E) or with (F) LatA treatment. Arrowheads show zones of Cdc42-rit^C^-GFP accumulation. Examination of individual focal planes showed that all of these zones are at the cell cortex. (G) Time lapse images of maximum projection of Cdc42-rit^C^-GFP fluorescence. Arrowheads show simultaneous bud emergence. Arrow shows bud emergence at a site distal to previous bud. The double-rim appearance of these images is due to under-sampling along the *z*-axis to preserve fluorescence. Time is shown in minutes. (H) Calcofluor-stained *wt* and *cdc42-rit* ^*C*^ cells, showing axial placement of bud scars in wt but disconnected bud scars in *cdc42-rit* ^*C*^. (I) Quantification of images as in (H). Most wild-type cells show clustered bud scars, whereas a large fraction of *cdc42-rit* ^*C*^ mutant cells display at least one scar or a group of scars disconnected from the others. Bars = 5 μm.

Our observations that Cdc42 polarizes independently of GDI and actin-based trafficking conflict with data in *S*. *cerevisiae* in which simultaneous disruption of GDI and actin blocks GFP-Cdc42 recycling and polarization [23,25]. We tested whether targeting of Cdc42 to the plasma membrane by an amphipathic helix would permit cell polarization and viability also in *S*. *cerevisiae* (Fig. 7A). We replaced the endogenous cdc42 gene in a diploid strain with a *cdc42-rit* ^*C*^ or a *cdc42-rit* ^*C*^ *-GFP* allele. Sporulation yielded four viable spores, of which two grew slowly and carried the mutant allele (Fig. 7B). Thus a Cdc42 allele directly targeted from the cytosol to the plasma membrane independently of GDI also confers viability in the budding yeast.

Cdc42-rit^C^-GFP efficiently polarized in haploid mutant cells, but often accumulated at two or more sites simultaneously (Fig. 7C–G), even upon actin disruption (Fig. 7F). A large fraction of these cells formed multiple buds or aberrant growth projections, which could grow concurrently (Fig. 7C–G). Examination of simultaneous double bud formation on time-lapse movies showed 45 events in 250 cells. Finally, these cells formed an abnormal budding pattern, budding at random locations, suggesting override of the normal landmarks at the previous bud scar (Fig. 7G–I) [62]. These data are entirely consistent with our results in the fission yeast and suggest that Cdc42 can also polarize to naïve sites at the plasma membrane independently of GDI and vesicle-mediated transport in the budding yeast.

## Discussion

### A Functional Fluorescently Tagged Cdc42 Allele

Living systems are able to spontaneously break symmetry and self-organize in ordered patterns. These patterns generally reflect the steady state of a dynamic protein flux. Thus, live fluorescently tagged alleles have become indispensable experimental tools. However, for small, highly conserved proteins that have multiple binding partners, it can be challenging to preserve functionality of the tagged molecule.

The highly conserved polarity regulator Cdc42 GTPase is one such small protein. N-terminal GFP fusions previously used to derive much of our knowledge on Cdc42 localization and dynamics compromise Cdc42 functionality and localization in yeasts [18,23,27]. N-terminal GFP-Cdc42 fusions have also been abundantly used in more complex eukaryotic organisms to derive information about Cdc42 localization and dynamics [63–68]. We note, however, that in these systems, functionality is more difficult to test.

We present here an improved internally tagged version of Cdc42 and its use in revealing new biology of this important polarity factor. We note that, besides the position of the fluorescent marker, its folding properties need to be taken into consideration, as use of mCherry or sfGFP, but not GFP, which folds considerably slower [39,40], resulted in functional fusions. All functional tests conducted here indicate these sandwich fusions do not compromise Cdc42 function, though we cannot rule out that phenotypes may be revealed in other, more sensitive backgrounds. Our approach is, in principle, generally applicable for Cdc42, or indeed for any small GTPase, in all organisms, though the specific site for fluorescent protein insertion will need to be carefully selected and tested.

### Cdc42 Polarization Independent of GDI and Vesicle Trafficking

The ability of Cdc42 to spontaneously polarize—i.e. to display local zones of accumulation—relies on positive feedback mechanisms. It has been proposed in the budding yeast to depend on two Cdc42 recycling routes from and to the plasma membrane: GDI-mediated extraction and trafficking on vesicles. In fission yeast, these two recycling routes very likely exist: Cdc42 is a probable GDI substrate because GDI binds Cdc42 [49], and GDI deletion causes modest reduction in Cdc42 dynamic turnover at cell sides. However, GDI deletion does not overtly affect the ability of Cdc42 to polarize in spores or vegetative cells. Cdc42 may also traffic on vesicles since it is detected on secretory vesicles upon block in exocytosis. Yet, short-term pharmacological treatment blocking vesicle trafficking causes no or very modest changes in Cdc42 dynamics. We note, however, that the actin cytoskeleton plays an important role in the longer-term stability of the polarized zone, which progressively diminishes at cell poles and spontaneously re-appears on cell sides upon sustained LatA treatment [42]. This may be similar to observations in the budding yeast, in which actin disruption causes flickering of the polarity patch and prolongs naturally observed oscillations [20,26]. We conclude that the actin cytoskeleton (and thus polarized vesicle transport and endocytosis) does not by itself significantly modify the dynamics of Cdc42 at the site of polarity, but plays a role in restraining the zone of Cdc42 activity to a stable location.

Importantly, we now provide extensive evidence that Cdc42 polarizes spontaneously even when both recycling routes are impaired. First, FRAP experiments show only modest effect on Cdc42 turnover rates upon combined GDI deletion and pharmacological disruption of vesicle trafficking. Second, Cdc42 can spontaneously polarize in spores lacking GDI and F-actin. Third, Cdc42-rit^C^ locally accumulates and supports polarized cell growth in multiple instances of spontaneous polarization in fission yeast: spore germination, protoplast polarization, or at cell sides in absence of the Tea1 landmark or upon long-term actin disruption. This allele localizes to the plasma membrane independently of GDI and vesicle trafficking: indeed, Cdc42-rit^C^ is likely not a substrate for the GDI because Cdc42 requires prenylation to bind Rdi1 in fission yeast [49], Rit^C^ is targeted to the plasma membrane directly from the cytosol [57], and we also show that Cdc42-rit^C^ does not accumulate on secretory vesicles. Finally, we show that this same allele, as sole Cdc42 copy, polarizes and supports polarized growth in *S*. *cerevisiae*. Here again, Cdc42-RitC likely breaks symmetry spontaneously, as the budding pattern is random, suggesting it polarized independently of the normal landmarks. We conclude that Cdc42 dynamics independent of GDI-extraction or vesicle trafficking play a key role in Cdc42 polarization.

This conclusion conflicts with the previous observation in *S*. *cerevisiae* that Cdc42 polarization is lost upon simultaneous GDI deletion and disruption of vesicle trafficking [23,25]. One explanation may lie in the use of the partially functional GFP-Cdc42 allele in previous dissection of Cdc42 dynamics. Another explanation may be that, in contrast to our engineering of a Cdc42 allele with a distinct membrane-targeting mode, GDI deletion or disruption of vesicle trafficking are likely to affect many other proteins besides Cdc42, and may thus have off-target effects. In any case, our results demonstrate that Cdc42 local accumulation can occur independently of proposed positive feedback on Cdc42 delivery and should incite re-examination of Cdc42 polarization models and of their dynamic parameters.

### Roles of Cdc42 Activity and Lateral Diffusion in Cdc42 Polarization

Cdc42 polarization (i.e. its local accumulation) and the polarization of its activity, as detected by the localization of a Cdc42-GTP reporter, are coincident in all cells examined to date (with the notable exception of the near-immobile Cdc42-psy1^TM^), suggesting that both events are intimately linked. Indeed, we provide very strong evidence that the local levels of Cdc42 robustly correlate with its local activity level both within wild-type cells and in a range of mutants. As the molecular role of some of the deleted proteins is to activate Cdc42 (e.g., Scd1), we can infer that Cdc42 local activation causes its accumulation. More convincingly, we show that Cdc42 local activation, in the Cdc42-psy1^TM^ allele, can occur without its local accumulation. This is likely due to the almost immobile behavior of this trans-membrane Cdc42 allele. Thus Cdc42 accumulation occurs as a result of its activation.

So how does Cdc42 accumulate at sites of activity? Our results provide strong evidence that active Cdc42-GTP moves more slowly at the membrane than inactive Cdc42-GDP. Indeed, Cdc42 turnover at cell sides, where absence of CRIB indicates that Cdc42 is in its GDP-bound form, is fast, with FRAP and FCS estimates of lateral diffusion constants of the order of 0.2 μm^2^/s. By contrast, zones of Cdc42 activity, either at cell tips or ectopically at cell sides, show significantly slower FRAP dynamics, and we observe a strong correlation between the levels of Cdc42 activity at the cell tip and Cdc42 FRAP halftime in a range of mutants. In addition, a GTP-locked Cdc42 allele also showed slow FRAP dynamics. Our interpretation is that Cdc42-GTP is significantly less mobile with estimates of diffusion rates at least 10-fold slower than Cdc42-GDP. This slower diffusion may be due to Cdc42-GTP forming large complexes with GEFs and effectors, multimerizing, or inducing the formation of membrane microdomains. In summary, Cdc42-GTP activation renders Cdc42 less mobile and leads to its accumulation.

We note that non-uniform Cdc42 dynamics were also observed in the budding yeast [22]. However, use of GTP- and GDP-blocked GFP-Cdc42 alleles, both of which showed dramatically reduced turnover, led to the different conclusion that Cdc42 hydrolysis cycle is required for its dynamic turnover [20,22].

Though exchange of Cdc42 between plasma membrane and cytosol likely participates, our results suggest that differential lateral diffusion of Cdc42-GTP and Cdc42-GDP contributes to Cdc42 polarization. A positive feedback mechanism acting on Cdc42 activation cycle, where a GEF is recruited by active Cdc42 [10–12,69], would lead to activation and thus “capture” of laterally diffusing Cdc42-GDP by Cdc42-GTP. This is, in principle, similar to previously proposed Turing-type mechanisms [13], but with the fast diffusing Cdc42-GDP component residing on the membrane rather than in the cytoplasm. Thus, lateral diffusion may play a positive role by providing material for polarization.

### Does Cdc42 Accumulation at the Polarized Zone Matter?

The relative depletion of Cdc42-psy1^TM^ from zones of local activation at cell tips, where it is more dynamic than at cell sides, is consistent with the idea that material for the cell tip normally derives from the cell sides. More strikingly, this finding raises the question of whether Cdc42 accumulation matters for spontaneous polarization. The synthetic phenotype observed with deletion of the Tea1 landmark suggests that local activation in absence of Cdc42 accumulation may be largely driven by pre-localized activators. However, the dynamic zones of Cdc42 activity that form on cell sides upon long-term LatA treatment reveal some level of spontaneous polarization in absence of Cdc42 enrichment. One prediction from these data is that positive feedback mechanisms acting on Cdc42 activation cycle, coupled to mechanisms preventing the propagation of Cdc42 activation to the entire plasma membrane [35], may be sufficient for symmetry breaking even without accumulation of Cdc42 itself. Future work should focus on the dynamic cycle of regulators of Cdc42 activity, in addition to Cdc42 itself.

### Singularity Versus Bipolarity

One important, yet unresolved, question is that of what underlies the ability of cells to polarize at single versus multiple zones. One general idea is that competition between polarized zones for a limiting factor allows one zone to win over the others, resulting in singularity [35]. This competition is enhanced by negative feedback mechanisms that destabilize the polarized zone and lead to oscillations [26]. Bipolarity may then result from saturation at the first polarized zone, allowing the initiation of a second one [28].

How may the alteration of Cdc42 membrane interaction in Cdc42-rit^C^ and Cdc42-psy1^TM^ alleles promote multi-site polarization? These more stable Cdc42 alleles may lead to faster saturation of a limiting factor at the polarized zone, for instance, due to absence of Cdc42 accumulation in the case of Cdc42-psy1^TM^. These alleles could also alter the negative feedbacks that normally destabilize the polarization sites, for instance, by modifying the proposed dilution effect caused by incoming vesicles [21,35]. More generally, these more stable Cdc42 alleles may compromise the competition between polarization sites by slowing down Cdc42 recycling, in agreement with the role of the GDI in promoting singularity [23].

In summary, our dissection and re-engineering of Cdc42 interaction with the plasma membrane in two very distinct organisms demonstrate that proposed positive feedback mechanisms acting on Cdc42 delivery to the plasma membrane are not essential for spontaneous polarization, but may be critical for the emergence of a single site. The growth and division mode of *S*. *cerevisiae* makes it highly dependent on establishing a single bud [23], whereas the requirements of *S*. *pombe* for mono- or bipolarity are less clear. It will be interesting to investigate whether evolutionary constraints on singularity may have shaped the molecular regulation of Cdc42 dynamic turnover.

## Materials and Methods

### Strain and Plasmid Construction

Fission and budding yeast strains used in this study are listed in S1 Table.

Expression plasmids were made by PCR amplification of *cdc42* from genomic DNA and cDNA using primers osm1471 (5′-ccGTCGACatgcccaccattaagtgtgtcg) and osm1483 (5′-tttCCCGGGttacagtaccaaacactttgac). Products were digested with SalI and XmaI and the resulting 1,189 bp (gDNA) and 596 bp (cDNA) products were ligated with similarly treated pREP41(*leu1+*) yielding pSM1135 (pREP41-*cdc42*-cDNA) and pSM1139 (pREP41-*cdc42-gDNA*). The linker-SGGSACSGPPG- was inserted after codon for Q134 as follows. First, PCR was done on cDNA and gDNA with primer pairs to amplify the 5′ and 3′ end of *cdc42*. The 5′ part was amplified with osm1471 and osm1481 (5′-ccagagcatgcGGATCCgccagactgatgctggcgagctag) and the 3′ part with osm1482 (5′-tctggcggatccgcatgctctGGCGCGCCgggccatccccttacacatgagc) and osm1483. The two products were subjected to PCR stitching with addition of primers osm1471 and 1483. The product was digested with SalI and XmaI and the resulting 618 bp (cDNA) and 1,211 bp (gDNA) product was ligated to similarly treated pREP41 yielding plasmids pSM1136 (pREP41-*cdc42-linker-cDNA*) and pSM1140 (pREP41-*cdc42-linker-gDNA*). Fluorophore genes were then cloned into the linker site. meGFP and mCherry were amplified by PCR with primers pairs osm1394 (5′-ccGGATCCatggtgagtaaaggagaagaacttttcactgg) and osm949 (5′-cccGGCGCGCCtttgtatagttcatccatgcc) for meGFP, and osm1393 (5′-ccGGATCCatggtgagcaagggcgaggaggataac) and osm947 (5′-cccGGCGCGCCcttgtacagctcgtccatgc) for mCherry. The products were digested with BamHI and AscI and the resulting 724 bp and 718 bp products, respectively, were ligated to similarly treated pSM1136 yielding pSM1137 (pREP41-*cdc42-GFP* ^*SW*^), pSM1136 yielding pSM1142 (pREP41-*cdc42-mCherry* ^*SW*^) and pSM1140 yielding pSM1138 (pREP41-*cdc42-cDNA-mCherry* ^*SW*^).

Integrative plasmids were made as follows. First, the 3′UTR of *cdc42* was amplified with primers osm1689 (5′-acgCATATGtttaacccctggttttctttcc) and osm1749 (5′-acgGTCGACgaatctcaactgggtttcgg) and the resulting 622 bp NdeI-SalI digested product was ligated to similarly treated pFA6a-kanMX yielding pSM1222 (pFA6a-3′UTR(*cdc42)*-kanMX). Next, the *cdc42-GFP* ^*SW*^ *-terminator* and *cdc42-mCherry* ^*SW*^ *-terminator* fragments from pSM1137 and pSM1138, respectively, were amplified with primers osm1471 and osm1686 (5′-acgACGCGTcttctaattacacaaattccg). The products were digested with SalI and MluI and ligated to similarly treated pSM1222 yielding plasmids pSM1223 (pFA6a-3′UTR(*cdc42)*-*cdc42-GFP* ^*SW*^-kanMX) and pSM1224 (pFA6a-3′UTR(*cdc42)*-*cdc42-mCherry* ^*SW*^-kanMX). The super folder GFP gene was amplified from pMaM4 [70] with primers osm2217 (5′-ccGGATCCtccaagggtgaagagctatttactgggg) and 2218 (5′-cccGGCGCGCCcttataaagctcgtccattccgtgag) and the product was digested with BamHI and AscI and the resulting 718 bp product was ligated to similarly treated pSM1224 yielding pSM1438 (pFA6a-3′UTR(*cdc42)*-*cdc42-sfGFP* ^*SW*^-kanMX).

Plasmids encoding retargeted or mutant *cdc42* were made as follows. For the prenylation defective mutant (noCAAX) PCR was done on template pSM1224 with primers osm1471 and osm2216 (5′-tttCCCGGGttactttgactttttcttgtgaggaacagg) and the 1,907 bp SalI-XmaI digested product was ligated to similarly treated pREP41 yielding pSM1364 (pREP41-*cdc42-noCAAX*). The integrative plasmid was made by subcloning the 891 bp BamHI-XmaI fragment from pSM1364 into pSM1438 yielding pSM1522 (pFA6a-3′UTR(*cdc42)*-*cdc42-mCherry* ^*SW*^-*noCAAX*-kanMX). For retargeted Cdc42 with the amphipathic helix rit^c^, PCR was done on template pSM1224 with primers osm1471 and 2219 (5′-aaaCCCGGGttaGCTAGCaggaacaggaggatcaagagcggctac) and the resulting1,292 bp SalI-XmaI digested product was ligated to similarly treated pREP41 yielding pSM1439 (pREP41-*cdc42-mCherry* ^*SW*^ *-noK-noCAAX*). Next, PCR was done with template pSM789 [57] and primers 2288 (5′-cctGCTAGCcacaagaaaaagtcaaagtgtcccttttttgagacatctgctgc) and 2289 (5′-ttaCCCGGGtcaagttactgaatctttcttcttccgg) and the resulting 213 bp NheI-XmaI digested product was ligated to the similarly treated pSM1439 yielding pSM1440 (pREP41-*cdc42-mChery* ^*SW*^ *-rit* ^*C*^). The integrative version was made by subcloning the 1,083 bp SalI-XmaI fragment from pSM1440 into similarly treated pSM1224 yielding pSM1442 (pFA6a-3′UTR(*cdc42)*-*cdc42-mCherry* ^*SW*^ *-rit* ^*C*^-kanMX). For the retargeted Cdc42 with transmembrane domain of tSNARE Psy1, PCR of gDNA with primers 2283 (5′-cctGCTAGCagagcagctcgtaagaaaaagtgg) and 2284 (5′-ttaCCCGGGtcaatgtctattgccaagaacagg) was done and the resulting 120 bp NheI-XmaI digested product was ligated to similarly treated pSM1439 yielding pSM1441 (pREP41-*cdc42-mCherry* ^*SW*^ *-psy1* ^*TM*^). The integrative version was made by subcloning the 990 bp BamHI-XmaI fragment from pSM1441 into the similarly treated pSM1224 yielding pSM1443 (pFA6a-3′UTR(*cdc42)*-*cdc42-mCherry* ^*SW*^ *-psy1* ^*TM*^-kanMX).

Plasmids pSM1223 (pFA6a-3′UTR(*cdc42)*-*cdc42-GFP* ^*SW*^-kanMX), pSM1224 (pFA6a-3′UTR(*cdc42)*-*cdc42-mCherry* ^*SW*^-kanMX), pSM1438 (pFA6a-3′UTR(*cdc42)*-*cdc42-sfGFP* ^*SW*^-kanMX), pSM1522 (pFA6a-3′UTR(*cdc42)*-*cdc42-mCherry* ^*SW*^-*noCAAX*-kanMX), pSM1442 (pFA6a-3′UTR(*cdc42)*-*cdc42-mCherry* ^*SW*^ *-rit* ^*C*^-kanMX) and pSM1443 (pFA6a-3′UTR(*cdc42)*-*cdc42-mCherry* ^*SW*^ *-psy1* ^*TM*^-kanMX) were linearized with SalI and transformed into diploid *S*. *pombe* cells. Integration was confirmed as described in S1 Fig Southern analysis was performed as follows. A probe was prepared using the random DIG labeling kit (Roche) and the 1,028 bp PCR product of gDNA with osm1471 and osm1481. Resolved genomic DNA digested with XbaI was probed along with controls SalI-XmaI and SalI digested pSM1139 (pREP41-*cdc42-gDNA*). Binding was detected using anti-DIG antibody and detected by chemoliminescence (Roche).

Plasmid pSM1358 (pREP41-*cdc42-Q61L-mCherry* ^*SW*^) was made by site directed mutagenesis using template pSM1142 and primers osm1983 (5′-cttggtttattcgataccgctggt**ctc**gaggattatgatcgcttgcg) and osm1984 (cgcaagcgatcataatcctc**gag**accagcggtatcgaataaaccaag) (codon change is underlined).

Replacement of prenylation sequences with the amphipathic helix rit^c^ in *S*. *cerevisiae* was done by a PCR-based targeting approach. First, the yeast codon-optimized *rit* ^*C*^ sequence was synthesized by Eurofins Genomics. The 195 bp SalI-XmaI digested *rit* ^*C*^ coding sequence was subcloned into similarly digested pFa6a-kanMX yielding pSM1534 (pFA6a-*rit* ^*C*^ *-kanMX*). Then, the 210 bp SalI-Asc1 digested fragment from pSM1534 was subcloned into pFA6a-GFP-term-KanMX yielding pSM1571 (pFA6a-*rit* ^*C*^ *-term-kanMX*). For tagging *cdc42* with *rit* ^*C*^ *-GFP*, PCR was performed with template pSM1534 and primers osm2688 (5′-cagGTCGACtgtccattttttgagac) and 2689 (5′-aaaCCCGGGagtaacagaatccttcttcttacgg) and the resulting digested 192 bp SalI-XmaI product was ligated to similarly treated pFA6a-GFP-term-KanMX yielding pSM1572 (pFA6a-*rit* ^*C*^ *-GFP-term-kanMX*). Plasmids pSM1571 and pSM1572 were used as templates for PCR with primers osm2625 (5′-caacgcggtttgaagaatgtattcgatgaagctatcgtggccgccttggagcctcctgttatcaagaaaagtaaaaaatgtccattttttgagacttctgc) and osm2949 (5′-taataaaaggataggaaggtgtatatataagttaattttagatatagattaagaaaagatgggcatatactaatatgagaattcgagctcgtttaaac) (*cdc42* homology underlined) to amplify product and transformed into diploid *S*. *cerevisiae* strains. Integration was confirmed by diagnostic PCR and diploids were sporulated to isolate haploid progeny with the *cdc42-rit* ^*C*^ *(-GFP)* allele as sole copy of *cdc42*.

### Growth Conditions, Pharmacological Inhibitors, and Sample Preparation

For *cdc42* temperature sensitivity complementation analysis cells were grown in EMM media supplemented with thiamine but without leucine to log phase at 25°C, washed three times in EMM without leucine and incubated overnight at 25°C in the same media. Cells were then diluted 10-fold and spotted onto EMM plates without leucine and incubated at indicated temperatures. Tetrads were dissected onto YE5S (*S*. *pombe*) or YPD (*S*. *cerevisiae*) and germinated at 25°C. For imaging analysis, cells were grown to log phase for at least 36 h by consecutive dilution of cultures in EMM at 25°C.

Imaging of calcofluor (Fig. 1F and 6C–E) and transmitted light (Fig. 7C) samples were done by placing cells on glass slides. All other cells were imaged on EMM, YE, or SD 2% agarose pads (SD for *S*. cerevisiae) and sealed with VALAP. LatA (Enzo Life Sciences, T-119) in DMSO was used at final concentration of 200 μM, except for imaging of spore outgrowth and *S*. *cerevisiae*, where it was used at 100 μM. Cells treated with Brefeldin A (Sigma, B7651) in ethanol were incubated in 100 μg/ml of drug for 1 h before imaging. Drugs were added directly to molten agarose before preparation of pads. Drug efficacy was monitored by mixing small amounts of control cells expressing the actin marker CDH-GFP (LatA) or the Arf GEF Sec71-GFP (BfA) directly into the imaging field and verifying marker mislocalization. Depletion of Sec8 from the *nmt81-sec8* strain was done by diluting cells grown in EMM without thiamine into EMM supplemented with 0.25 μg/ml thiamine and grown for 20 h at 25°C.

For calcofluor staining of *S*. *cerevisiae* bud scars, cells were diluted from a pre-culture and grown overnight before staining with calcofluor and imaging.

For the re-feeding experiments of *tea1*Δ strains, cells were grown to starvation in YE5S liquid media for 4 d and diluted 1:50 into YE and stained with calcofuor after 4 h. Imaging of G1 arrested *cdc10-v50* strains was done by diluting precultures in EMM at 25°C into EMM and grown at 36°C for 6 h before staining with calcofluor.

For FRAP imaging of Cdc42-GTP in S3 Fig, *cdc42-sfGFP* ^*SW*^ *-kanMX* cells harboring plasmids pSM1139 (pREP41-*cdc42-mCherry* ^*SW*^) or pSM1358 (pREP41-*cdc42-Q61L-mCherry* ^*SW*^) were grown as follows. Cells were grown overnight in EMM-AU supplemented with thiamine and washed three times in EMM-AU lacking thiamine. Cells were diluted to OD_600_ = 0.05 and grown for 18 h before imaging.

### Microscopy

Spinning disk confocal images were acquired on a Leica DMI4000B or DMI6000SD inverted microscope equipped with an HCX PL APO 100X/1.46 numerical aperture (NA) oil objective and a PerkinElmer Confocal system. This system uses a Yokagawa CSU22 real-time confocal scanning head, solid-state laser lines and a cooled 14-bit frame transfer EMCCD C9100-50 camera (Hamamatsu) and is run by Volocity (PerkinElmer). Time-lapse microscopy (Figs. 6A,B, 7G and S6D–E) was performed on a DeltaVision platform (Applied Precision). This platform is composed of a customized Olympus IX-71 inverted microscope equipped with a UPlan Apo 100X/1.4 NA oil objective, a CoolSNAP HQ2 camera (Photometrics), and an Insight SSI 7 color combined unit illuminator. Calcofluor and transmitted light microscopy was performed on either the DeltaVision system or a Leica DMI400B microscope equipped with a HCX PL Fl 63X/1.25 NA objective. The FRAP data acquired for analysis in Fig. 4C,D and S5A–F was acquired on a Zeiss Axio Observer. Z1 inverted microscope equipped with a LSM 710 scan head, a PL APO 100X/1.4 NA oil DIC objective and Argon multiline 458/488/514 nm (25.0 mW) (Lasos) and 561 nm (20.0 mW) laser.

### Fluorescence Image Quantification

Quantification of Cdc42-mCherry^SW^ and CRIB distribution shown in Fig. 2B–D, 5F, 5H, S2 was done by using the sum projection of five consecutive images. The intensity of a 3-pixel-wide segment was collected from at least 20 tips for both red and green channels. The tips were centered around the maximum pixel values for the CRIB channel and subsequently split into two half tips. Enrichment values were derived by dividing the maximum intensity value by the average intensity of the final 1 μm region.

### Spore Imaging

Spores were prepared from mating mixtures by overnight digestion in Glusulase at 25°C followed by three washes with water. The spores generated from *cdc42-mCherry* ^*SW*^ *-rit* ^*C*^ and the wild-type control crosses (Fig. 6B) the spores were enriched by addition to Percoll (Sigma) and centrifugation at 10K for 10 min. Dense spores were collected from the bottom fraction. For fluorescence imaging of germinating spores in Fig. 4B spores were incubated in YE media at 25°C for 3 h before placed onto YE 2% agarose pads and sealed with VALAP before imaging.

### Fluorescence Recovery after Photobleaching (FRAP)

FRAP data from Figs. 3A,B–E, 4A,E, 5E,I and S3C was gathered with a Photokinesis module on the spinning disk confocal system described above. A 0.9 μ m region was bleached following two pre-bleach acquisitions and recovery was followed at regular intervals except those for GFP-Psy1 and Cdc42-mCherry^SW^-psy1^TM^ where intervals increased over time. Analysis of FRAP was done as previously described [71]. For S3 Fig the green and red channels were simultaneously bleached and the fluorescence in each channel was monitored. The significance of differences in FRAP values is depicted in figures with asterisks as follows; n.s. = *p* > 0.05, * = *p* ≤ 0.05, ** = *p* ≤ 0.01, *** = *p* ≤ 0.001, **** = *p* ≤ 0.0001.

The FRAP experiments described in Figs. 4C and S5A,C were performed by bleaching a cortical region at the cell side or a cortical region that included half of the cell tip and part of the cell side with the Zeiss laser scanning system described above. The contour of the cell membrane was measured with JFilament, an ImageJ plugin for segmentation and tracking of 2-D and 3-D filaments in fluorescence microscopy images (S5A Fig) [72]. This program was used to fit a closed smooth active contour to the cell boundary of an image of the cell prior to bleaching. We used settings that attract the contour to image regions with a local maximum intensity gradient. The membrane intensity was calculated as a function of distance along the contour and time by integrating the intensity within a band around the contour that included the cell membrane (typically 3 pixels), in the direction normal to the contour, using Boundary Kymograph in JFilament. To correct for continuous photobleaching during image acquisition, the intensities at each time were normalized to the average intensity at the unbleached cell side, after subtracting the out-of-cell background. With this normalization method, the profiles at times of order 30 s after bleaching recovered close to the pre-bleach profile, even when continuous photobleaching caused the overall intensity to drop by 50% over that time.

The normalized intensity profiles *I*(*x*,*t*) at the cell sides (Fig. 4D,E) were fitted to the following equation that represents recovery by diffusion with diffusion coefficient *D* and uniform exchange with the cytoplasm with time constant *τ*, along an approximately flat membrane:

$$I(x,t)=I_{0}-C\;e^{-t/\tau }e^{-{\left(x-x_{0}\right)}^{2}/(4Dt+2\sigma_{0}^{2})}/[{\left(4\pi Dt+2\pi \sigma_{0}^{2}\right)}^{1/2}{\left(4\pi Dt+2\pi \sigma_{z}^{2}\right)}^{1/2}]$$

Here *x* is distance along the cell contour, *I*_0_ is the value of the uniform intensity prior to bleaching, *x*_0_ is the position of the center of the bleached region, *σ*_0_ gives the standard deviation of the initial bleached region along the cell contour that has an approximately Gaussian shape, *σ*_*z*_ = 0.8 − 1.5 μm is the estimated standard deviation of the bleached region in the *z* direction, and *C* is a constant describing the magnitude of the intensity dip in the bleached region. The above expression is obtained by evolving an initial 2-D bleached distribution with a Gaussian shape in each direction with the 2-D free diffusion propagator, multiplied by an exponential representing uniform cytoplasmic exchange.

To obtain estimates for *D* and *τ*, the recovery curves of narrow bleached regions were first fitted by assuming that they are dominated by diffusion and setting the exponential term dependent on *τ* to unity (Fig. 4E). The obtained value of *D* was then used to fit recovery curves in wider bleached regions and obtain an estimate for *τ* (Fig. 4D). The above procedure is self-consistent since the resulting value of *τ* is large enough to justify neglecting it for the recovery of narrow bleach regions. The error bar of the reported *D* and *τ* values is obtained by observing the range of values that allow fits going through the data, within the experimental noise and the range of values of σ_z_. Larger (smaller) values of D and τ are obtained for larger (smaller) σ_z_.

To obtain an upper bound for the diffusion coefficient of Cdc42-GTP, the distance *d* between the maximum and half-maximum of the intensity profile within the tip bleached region was measured versus time (S5D Fig). This value was used to calculate the standard deviation σ = 085 *d* of an equivalent 1-D Gausian diffusion propagator. A diffusion coefficient *D*_slow_ was then estimated by the setting the slope of the graph in S5E Fig to 2*D*_slow_.

### Protoplast Recovery Assay

Protoplasts were generated as described previously with modifications (Flor-Parra et al. 2014. Yeast). Cells grown in EMM were loaded into chambers of microfluidic plates from CellASIC ONIX (Miilipore) prewashed with EMM. Cells were then washed with E buffer (50mM NaCitrate, 100 mM NaPhosphate pH5.6, 1.2 M Sorbitol) for 10 min followed by cell wall digestion by flowing in 0.1 g/ml Lallzyme MMX (Lallemand) in E buffer for 15 min. The resulting protoplasts were allowed to recover by flowing in EMM containing 1 M Sorbitol and cells were imaged every 20 min.

### Fluorescence Correlation Spectroscopy (FCS)

Fluorescence correlation spectroscopy experiments were performed on a LSM 510 Meta laser scanning microscope equipped with a ConfoCor 3 unit (Carl Zeiss MicroImaging GmbH). A 40x/1.2NA water-immersion objective focused the 488 and 561 nm excitation laser beams to a diffraction-limited spot and collected the emitted fluorescence light. The location of the excitation/observation volume on different parts of the yeast was adjusted as close as possible to the plasma membrane with the help of a galvano mirror and an acousto-optical tunable filter (AOTF), which fine-tuned the incident irradiation. Fluorescence time traces were recorded for 10x10 seconds and a built-in photon correlator analyzed the fluorescence fluctuations to generate autocorrelation curves:

$$G(\tau )=\frac{\langle F(t)\cdot F(t+\tau )\rangle }{{\langle F(t)\rangle }^{2}}$$

where <…> denotes time average, *F(t)* the fluorescence signal and *τ* the delayed time, also called lag time.

The autocorrelation curves were subsequently fitted with diffusion models using a Marquardt algorithm on Igor Pro software (WaveMetrics) to extract different parameters such as translational diffusion time *τ*_*D*_ and mean number of particles *N* in the observation volume as described in detail elsewhere [73]. Analysis of receptor diffusion on yeast membrane was performed by combining a three-dimensional diffusion of free proteins in cytosol and a two-dimensional diffusion of membrane receptors:

$$G\left(\tau \right)=1+\frac{1}{N}\left(F_{memb}{\left(1+\frac{\tau }{\tau_{Dmemb}}\right)}^{-1}+\left(1-F_{memb}\right){\left(1+\frac{\tau }{\tau_{Dcyt}}\right)}^{-1}{\left(1+\frac{\tau }{S^{2}\tau_{Dcyt}}\right)}^{-1/2}\right)$$

where *τ*_*memb*_ and τ_*cyt*_ are the diffusion times of the membrane receptor and free proteins, respectively, *S* is the structure parameter defined by the ratio of the axial and lateral axes of the observation volume and *F*_*memb*_ is the fraction of receptor. To take into account triplet state, the second term might be multiplied by:

$$\left(1+\frac{T}{1-T}e^{-\frac{\tau }{\tau_{T}}}\right)$$

where *T* is the triplet fraction and *τ*_*T*_ the triplet time.

Finally, diffusion coefficients *D* were calculated according to:

$$D=\frac{\omega_{xy}^{2}}{4\cdot \tau_{D}}$$

where the lateral beam waist radius *ω*_*xy*_ was determined measuring the translational diffusion time of a calibration dye of known diffusion coefficient.

## Supporting Information

S1 Data Raw data of graphs presented in the figures. (XLSX)

S1 Fig Controls for *cdc42* sandwich-tagged alleles. (A) Schematic of integrative plasmid recombination. Grey zones represent genomic non-coding regions. Thin lines represent integrative plasmid, including terminator (term). (B) Agarose gels of diagnostic PCR from genomics DNA prepared from wt and listed *cdc42* ^*SW*^ genomes. Binding sites for primers are shown above DNA boxes in A. PCR product lengths for each primer pair and genotype are listed on the right. (C) Southern blot of XbaI digested genomic DNA from wt (1), *cdc42-mCherry* ^*SW*^ *-kanMX* (2), *cdc42-GFP* ^*SW*^ *-kanMX* (3), SalI-XmaI digested pSM1139 (pREP41-*cdc42*) yielding 1.2 kb *cdc42* reading frame (4) and SalI-linearized pSM1139 yielding 9.9 kb linear fragment. Probe hybridization site and expected genomic DNA restriction fragment lengths are shown above and below DNA boxes in A, respectively. (D) Average cell length and width (top), septal position (middle), and percent cells growing mono- or bipolar (bottom) for indicated strains. (E) Medial spinning disk confocal section of Cdc42-sfGFP^SW^. Bar = 5 μm.
(TIF)

S2 Fig Correlation of CRIB and Cdc42 accumulation in wild-type cells. (A) Cdc42-mCherry^SW^ and CRIB-3GFP localization. Arrowheads indicate low-CRIB level cell ends. (B) Average profiles of fluorescence intensity along cortical traces for low- and high-CRIB cell ends. Bar = 5 μm.
(TIF)

S3 Fig Slow mobility of GTP-locked Cdc42^Q61L^-mCherry^SW^ allele. (A) Transmitted light images of cells after 36 h or repression or induction (− thiamine) of *cdc42* ^*Q61L*^ *-mCherry* ^*SW*^ from pREP41 plasmid. Bar = 5 μm. (B) Medial spinning disk confocal section of Cdc42-sfGFP^SW^ expressed from the endogenous promoter and Cdc42-mCherry^SW^ or *Cdc42* ^*Q61L*^ *-mCherry* ^*SW*^ expressed from pREP41 plasmid after 18 h of induction. Bar = 5 μm. (C) FRAP halftimes (t^1/2^) at cell tips and cell sides of Cdc42-mCherry^SW^ (left) or Cdc42^Q61L^-mCherry^SW^ (right) expressed from plasmids in strains expressing Cdc42-sfGFP^SW^ from the endogenous promoter. The FRAP halftimes of Cdc42-sfGFP^SW^ are also shown. Cdc42-mCherry^SW^ has no strong effect on the dynamics of Cdc42-sfGFP^SW^. By contrast, Cdc42^Q61L^-mCherry^SW^ shows slow dynamics and induces fast dynamics of Cdc42-sfGFP^SW^ at cell tips. *n* ≥ 10. The asterisks denote statistical significance in a Student’s *t* test when comparing the tips and sides of the endogenous *cdc42-sfGFP* ^*SW*^. n.s. = *p* > 0.05 * is *p* ≤ 0.05, ** is *p* ≤ 0.01, *** is *p* ≤ 0.001, **** is *p* ≤ 0.0001.
(TIF)

S4 Fig Minor or no phenotypic defects in *rdi1Δ* cells. (A) 10-fold serial dilutions of wt and *rdi1*Δ cells. (B) Growth curve of cells grown in EMM at 30°C. (C) Average length, width and septal position of wt and *rdi1*Δ cells grown in EMM at 30°C. (D) Cdc42-mCherry^SW^ and CRIB-3GFP localization in *rdi1*Δ cells. Bar = 5 μm. (E) Time-lapse images of Scd2-GFP and Cdc42-mCherry^SW^ localization in wild-type and *rdi1*Δ cells treated with LatA. Time is shown in minutes. T = 0 represents the first taken image, about 5 min after LatA addition. Arrowheads show examples of cell side accumulation of Scd2-GFP and Cdc42-mCherry^SW^. Bar = 5 μm.
(TIF)

S5 Fig Additional measurements of Cdc42 diffusion by FCS and FRAP. (A) Cdc42-mCherry^SW^ images before and after cortical bleach of half-tip and cell side. Red line shows active contour fitted to the cell boundary with JFilament. The intensity as function of distance along the membrane *s* is measured by integrating the intensity within a band of width 3 pixels around the contour. (B) Intensity profile along cell side versus time for cell in panel A, around the bleached region. A transient initial decrease in intensity is observed at the position of the black arrow, indicating diffusive redistribution of bleached Cdc42 across the cell tip. (C) Kymograph showing intensity around cell tip versus time for cell in panel A. (D) Measurement of half-maximum of the intensity profile within the bleached region versus time. (E) Fit of the width estimated in panel D to a 1-D uniform diffusion model. Broadening of the post-bleach intensity profile could involve diffusion of Cdc42-GDP from cell sides and local conversion to less mobile Cdc42-GTP, as well as diffusion of Cdc42-GTP itself. The diffusion coefficient calculated in this panel is an estimate of the upper limit to the diffusion coefficient of Cdc42-GTP (since larger diffusion coefficients should produce faster broadening). (F) Intensity profile along cell side versus time for *rdi1Δ* expressing Cdc42-mCherry^SW^ bleached along the cell side as in Fig. 4C. The intensity along the membrane was measured by fitting an active contour to the cell boundary and integrating the intensity within 3 pixels. Continuous lines show fit to a model of recovery with diffusion coefficient *D* and uniform cytoplasmic exchange with time constant *τ* (see Materials and Methods). (G) Normalized FCS autocorrelation curves of GFP at side (black), of Cdc42-mCherry^SW^–no-CAAX at side (violet) and tip (blue) and of GFP-CAAX at side (red) and green (tip). The specific extension of the GFP-CAAX curves to higher lag time values indicates this depends on prenylation. The curves were fitted with a 2-component diffusion model taking into account the triplet state (grey dashed lines). (H) Summarizing table of important parameters measured for Cdc42-mCherry^SW^ at side and tip locations. (I) Summarizing table of important parameters measured for diverse proteins as control at side and tip locations.
(TIF)

S6 Fig Cdc42 CAAX box is essential for function and Cdc42SW-psy1TM and Cdc42SW-ritC form zones at cell sides upon long-term LatA treatment. (A) Complementation of *cdc42-1625* temperature sensitivity by pREP41-based plasmids. (B) Representative images of haploid cells from wt/*cdc42-mCherry* ^*SW*^ *noCAAX*:*kanMX* diploid tetrad dissection. Inset in top and bottom panels show high magnification. Note *cdc42-mCherry* ^*SW*^ *noCAAX*:*kanMX* cells arrest growth following first division attempt, likely because of zygotic wt Cdc42 perdurance in the first cell cycle. (C) Average cell length and width (left), septal position (middle) and percent cells growing in a mono- or bipolar manner (right) for *cdc42* ^*SW*^ *-psy1* ^*TM*^ and *cdc42* ^*SW*^ *-rit* ^*C*^ strains (compare to S1D Fig). (D and E) Time-lapse images of Cdc42-mCherry^SW^–psy1^TM^ (D) or Cdc42-mCherry^SW^–rit^C^ (E) and CRIB-3GFP localization following treatment with LatA. Time is shown in minutes. T = 0 represents the first taken image, about 5 min after LatA addition. Arrowheads show examples of cell side accumulation of CRIB-3GFP and Cdc42-mCherry^SW^. (F) Calcofluor images of indicated strains in log phase. (G) 10-fold serial dilutions of indicated strains on YE. Bars = 5 μm.
(TIF)

S7 Fig Polarization of Cdc42-rit^C^ in spores and bipolar growth of *cdc42-rit* ^*C*^ and *cdc42-psy1* ^*TM*^ mutants. (A) Time lapse images of Cdc42-mCherry^SW^ and Cdc42-mCherry^SW^-rit^C^ fluorescence during spore outgrowth on YE. Arrowheads indicate site of fusion protein accumulation followed by polarized growth. Time is shown in minutes. Bar = 5 μm. (B) DIC images of indicated strains grown in microfluidic chambers with EMM. Red and blue lines represent cell growth at the old and the new cell end, respectively. Time is shown in h. Bar = 10 μm.
(TIF)

S1 Table Fission and budding yeast strains used in this study. (DOCX)

## Abbreviations

BFA: Brefeldin A
CRIB: Cdc42- and Rac-interactive binding
DIC: Differential interference contrast
EMM: Edinburgh minimal medium
FCS: fluorescence correlation spectroscopy
FRAP: fluorescence recovery after photobleaching
GDIs: Guanine nucleotide Dissociation Inhibitors
GEFs: Guanine nucleotide Exchange Factors
GFP: green fluorescent protein
LatA: Latrunculin A
PAK: p21-activated kinases
YE: yeast extract
wt: wild type
