# Supplementary figures and images for: Spontaneous Cdc42 Polarization Independent of GDI-Mediated Extraction and Actin-Based Trafficking
Source: PLoS Biol. 2015 Apr 2;13(4):e1002097. doi: 10.1371/journal.pbio.1002097 (PMC4383620; doi:10.1371/journal.pbio.1002097)

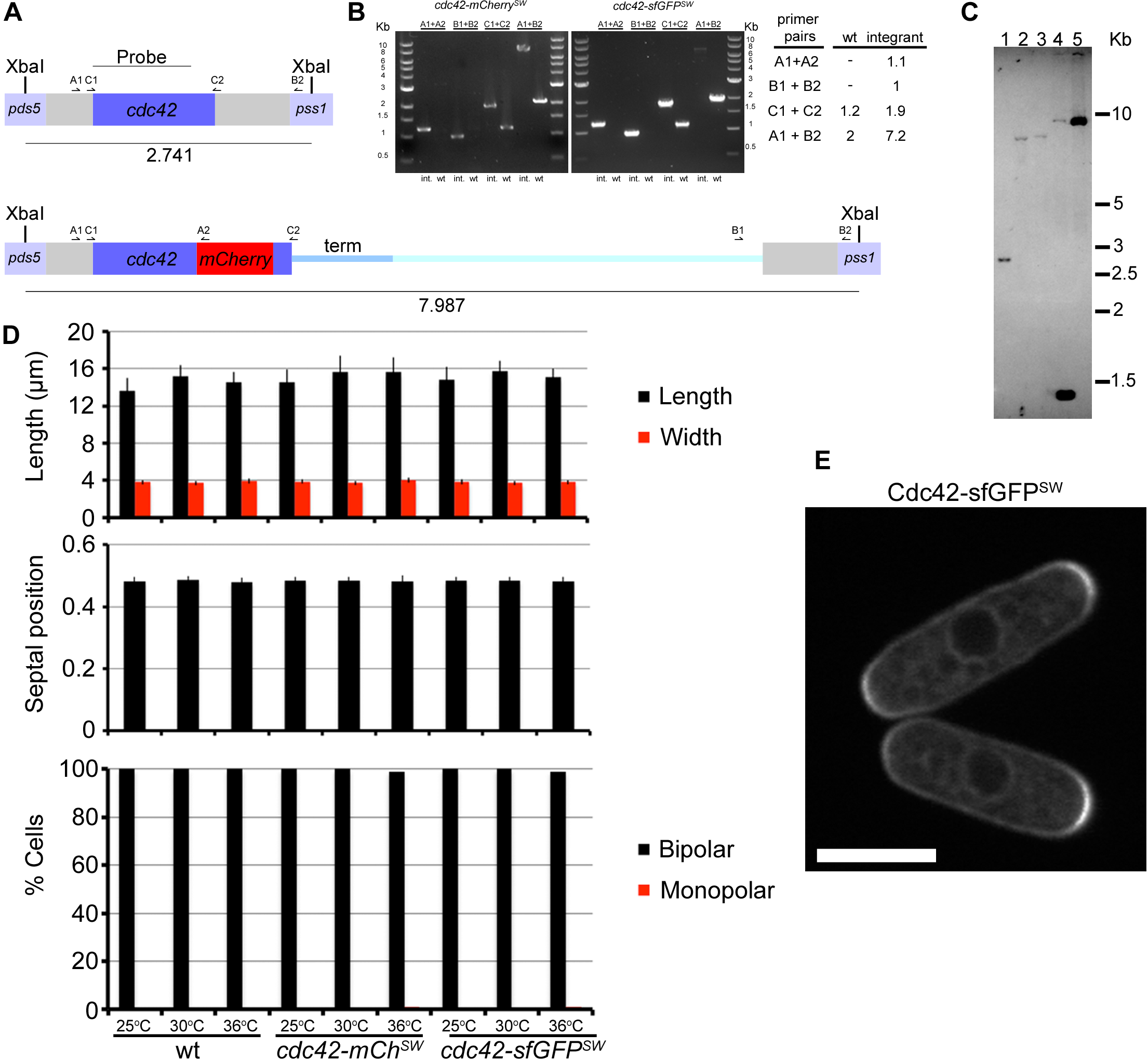

Supplement: S1 Fig — (A) Schematic of integrative plasmid recombination. Grey zones represent genomic non-coding regions. Thin lines represent integrative plasmid, including terminator (term). (B) Agarose gels of diagnostic PCR from genomics DNA prepared from wt and listed cdc42 SW genomes. Binding sites for primers are shown above DNA boxes in A. PCR product lengths for each primer pair and genotype are listed on the right. (C) Southern blot of XbaI digested genomic DNA from wt (1), cdc42-mCherry SW -kanMX (2), cdc42-GFP SW -kanMX (3), SalI-XmaI digested pSM1139 (pREP41-cdc42) yielding 1.2 kb cdc42 reading frame (4) and SalI-linearized pSM1139 yielding 9.9 kb linear fragment. Probe hybridization site and expected genomic DNA restriction fragment lengths are shown above and below DNA boxes in A, respectively. (D) Average cell length and width (top), septal position (middle), and percent cells growing mono- or bipolar (bottom) for indicated strains. (E) Medial spinning disk confocal section of Cdc42-sfGFPSW. Bar = 5 μm. (TIF) [file pbio.1002097.s002.tif]

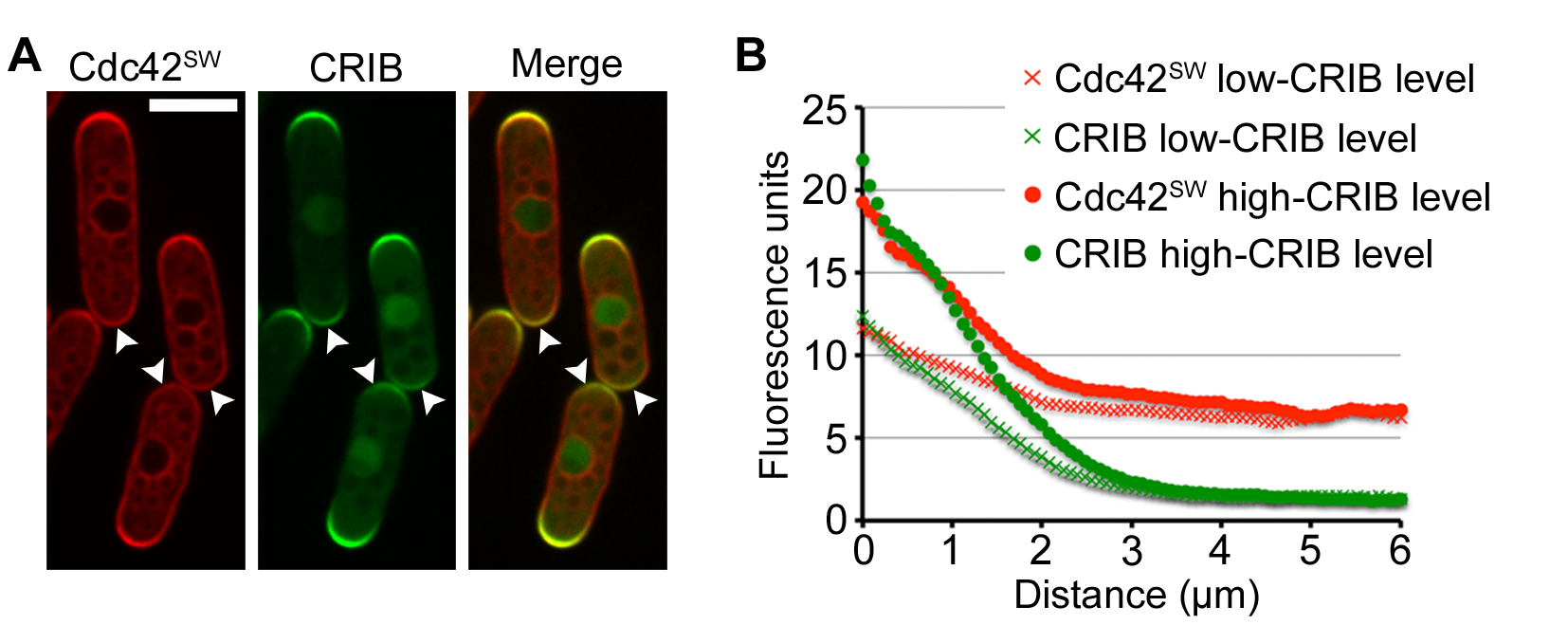

Supplement: S2 Fig — (A) Cdc42-mCherrySW and CRIB-3GFP localization. Arrowheads indicate low-CRIB level cell ends. (B) Average profiles of fluorescence intensity along cortical traces for low- and high-CRIB cell ends. Bar = 5 μm. (TIF) [file pbio.1002097.s003.tif]

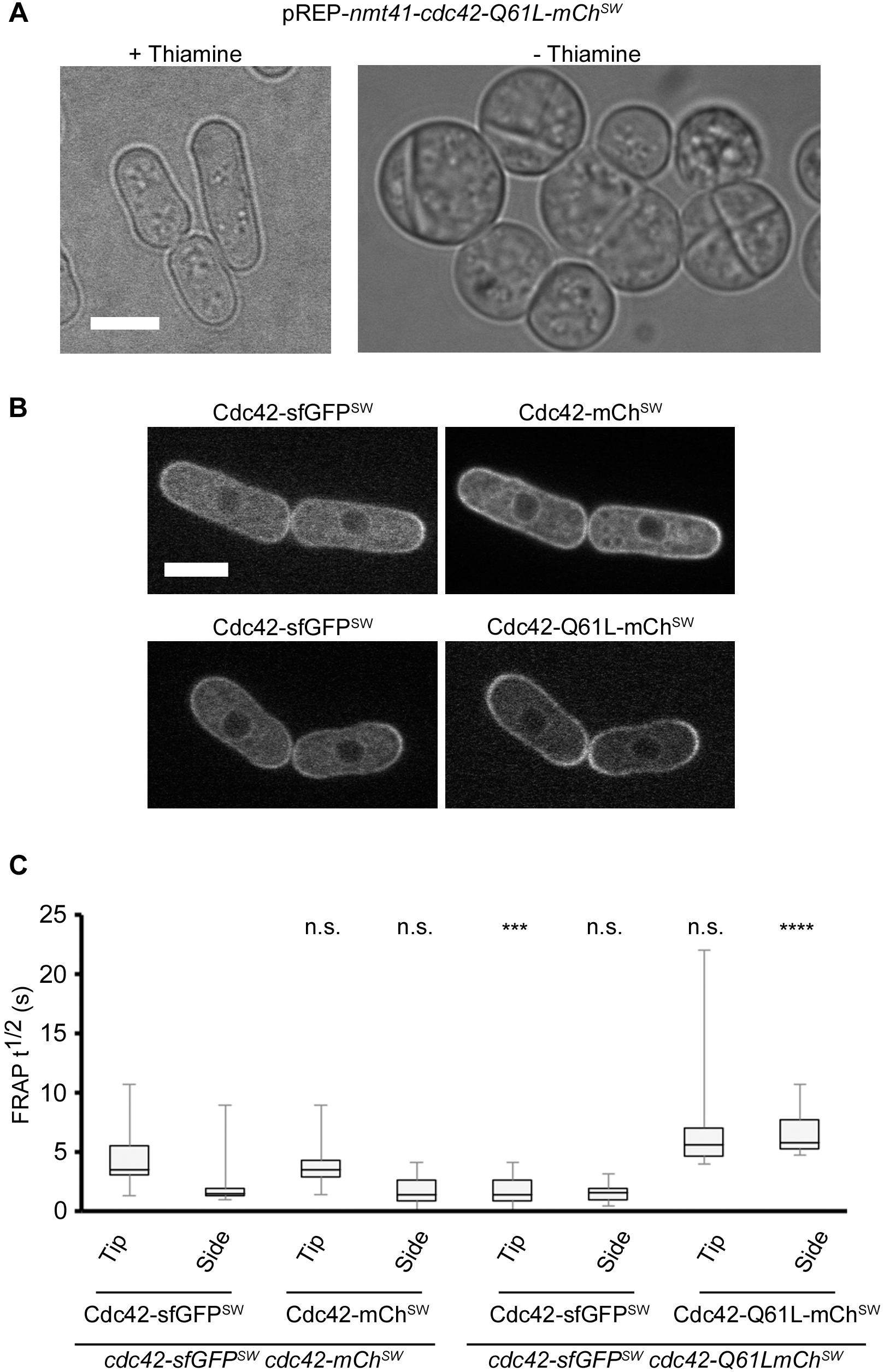

Supplement: S3 Fig — (A) Transmitted light images of cells after 36 h or repression or induction (− thiamine) of cdc42 Q61L -mCherry SW from pREP41 plasmid. Bar = 5 μm. (B) Medial spinning disk confocal section of Cdc42-sfGFPSW expressed from the endogenous promoter and Cdc42-mCherrySW or Cdc42 Q61L -mCherry SW expressed from pREP41 plasmid after 18 h of induction. Bar = 5 μm. (C) FRAP halftimes (t1/2) at cell tips and cell sides of Cdc42-mCherrySW (left) or Cdc42Q61L-mCherrySW (right) expressed from plasmids in strains expressing Cdc42-sfGFPSW from the endogenous promoter. The FRAP halftimes of Cdc42-sfGFPSW are also shown. Cdc42-mCherrySW has no strong effect on the dynamics of Cdc42-sfGFPSW. By contrast, Cdc42Q61L-mCherrySW shows slow dynamics and induces fast dynamics of Cdc42-sfGFPSW at cell tips. n ≥ 10. The asterisks denote statistical significance in a Student’s t test when comparing the tips and sides of the endogenous cdc42-sfGFP SW. n.s. = p > 0.05 * is p ≤ 0.05, ** is p ≤ 0.01, *** is p ≤ 0.001, **** is p ≤ 0.0001. (TIF) [file pbio.1002097.s004.tif]

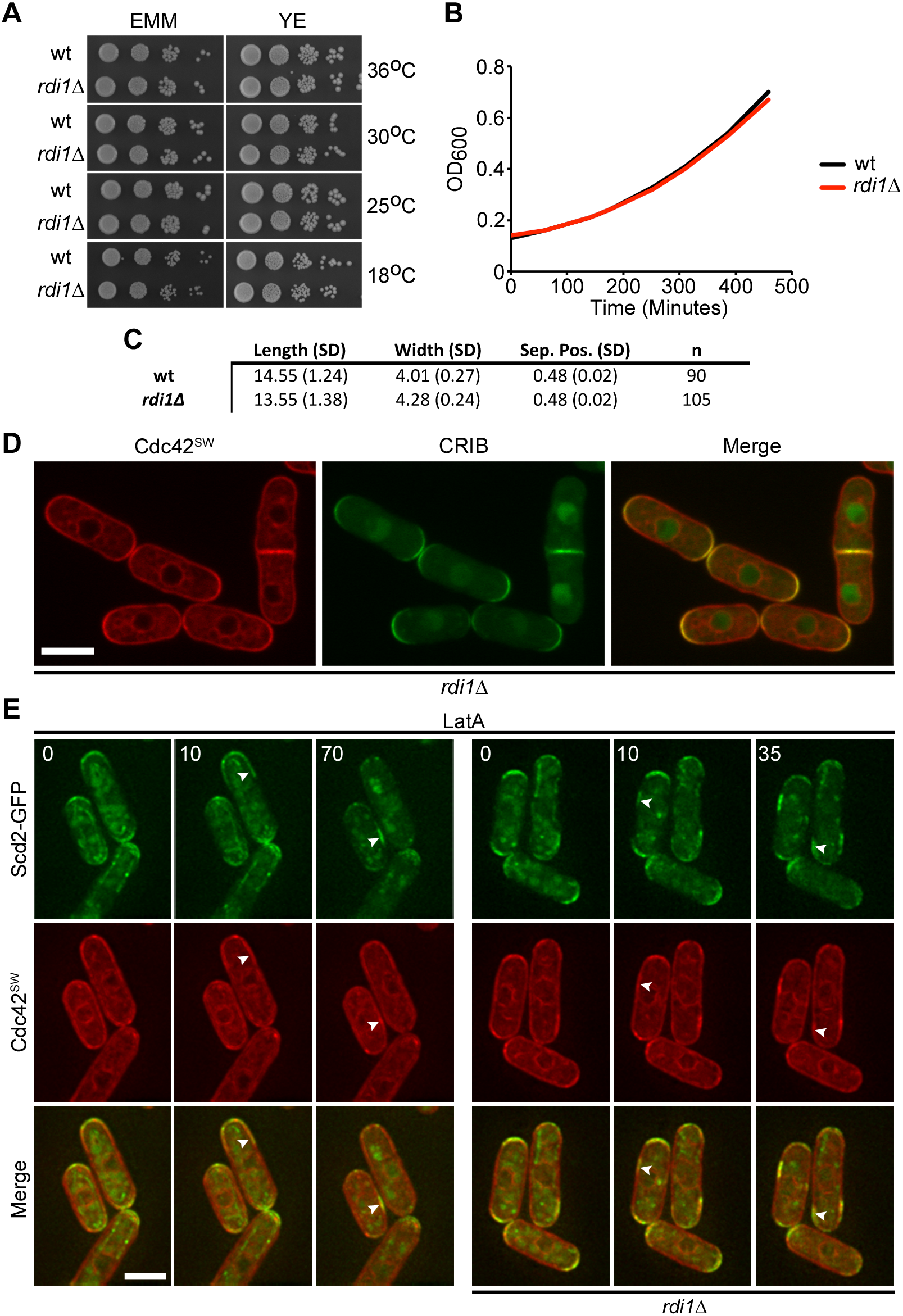

Supplement: S4 Fig — (A) 10-fold serial dilutions of wt and rdi1Δ cells. (B) Growth curve of cells grown in EMM at 30°C. (C) Average length, width and septal position of wt and rdi1Δ cells grown in EMM at 30°C. (D) Cdc42-mCherrySW and CRIB-3GFP localization in rdi1Δ cells. Bar = 5 μm. (E) Time-lapse images of Scd2-GFP and Cdc42-mCherrySW localization in wild-type and rdi1Δ cells treated with LatA. Time is shown in minutes. T = 0 represents the first taken image, about 5 min after LatA addition. Arrowheads show examples of cell side accumulation of Scd2-GFP and Cdc42-mCherrySW. Bar = 5 μm. (TIF) [file pbio.1002097.s005.tif]

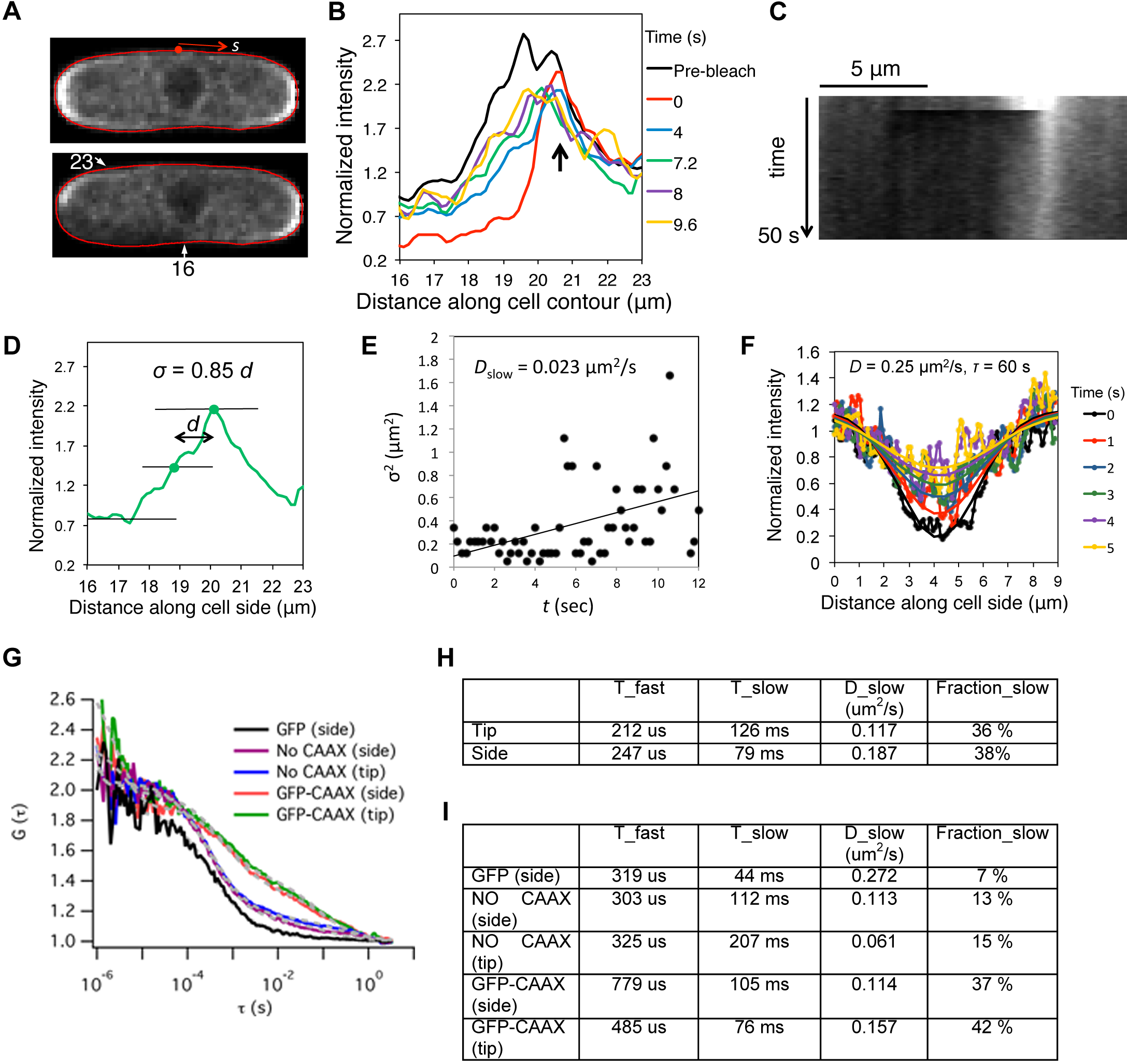

Supplement: S5 Fig — (A) Cdc42-mCherrySW images before and after cortical bleach of half-tip and cell side. Red line shows active contour fitted to the cell boundary with JFilament. The intensity as function of distance along the membrane s is measured by integrating the intensity within a band of width 3 pixels around the contour. (B) Intensity profile along cell side versus time for cell in panel A, around the bleached region. A transient initial decrease in intensity is observed at the position of the black arrow, indicating diffusive redistribution of bleached Cdc42 across the cell tip. (C) Kymograph showing intensity around cell tip versus time for cell in panel A. (D) Measurement of half-maximum of the intensity profile within the bleached region versus time. (E) Fit of the width estimated in panel D to a 1-D uniform diffusion model. Broadening of the post-bleach intensity profile could involve diffusion of Cdc42-GDP from cell sides and local conversion to less mobile Cdc42-GTP, as well as diffusion of Cdc42-GTP itself. The diffusion coefficient calculated in this panel is an estimate of the upper limit to the diffusion coefficient of Cdc42-GTP (since larger diffusion coefficients should produce faster broadening). (F) Intensity profile along cell side versus time for rdi1Δ expressing Cdc42-mCherrySW bleached along the cell side as in Fig. 4C. The intensity along the membrane was measured by fitting an active contour to the cell boundary and integrating the intensity within 3 pixels. Continuous lines show fit to a model of recovery with diffusion coefficient D and uniform cytoplasmic exchange with time constant τ (see Materials and Methods). (G) Normalized FCS autocorrelation curves of GFP at side (black), of Cdc42-mCherrySW–no-CAAX at side (violet) and tip (blue) and of GFP-CAAX at side (red) and green (tip). The specific extension of the GFP-CAAX curves to higher lag time values indicates this depends on prenylation. The curves were fitted with a 2-component diffusion model taki [file pbio.1002097.s006.tif]

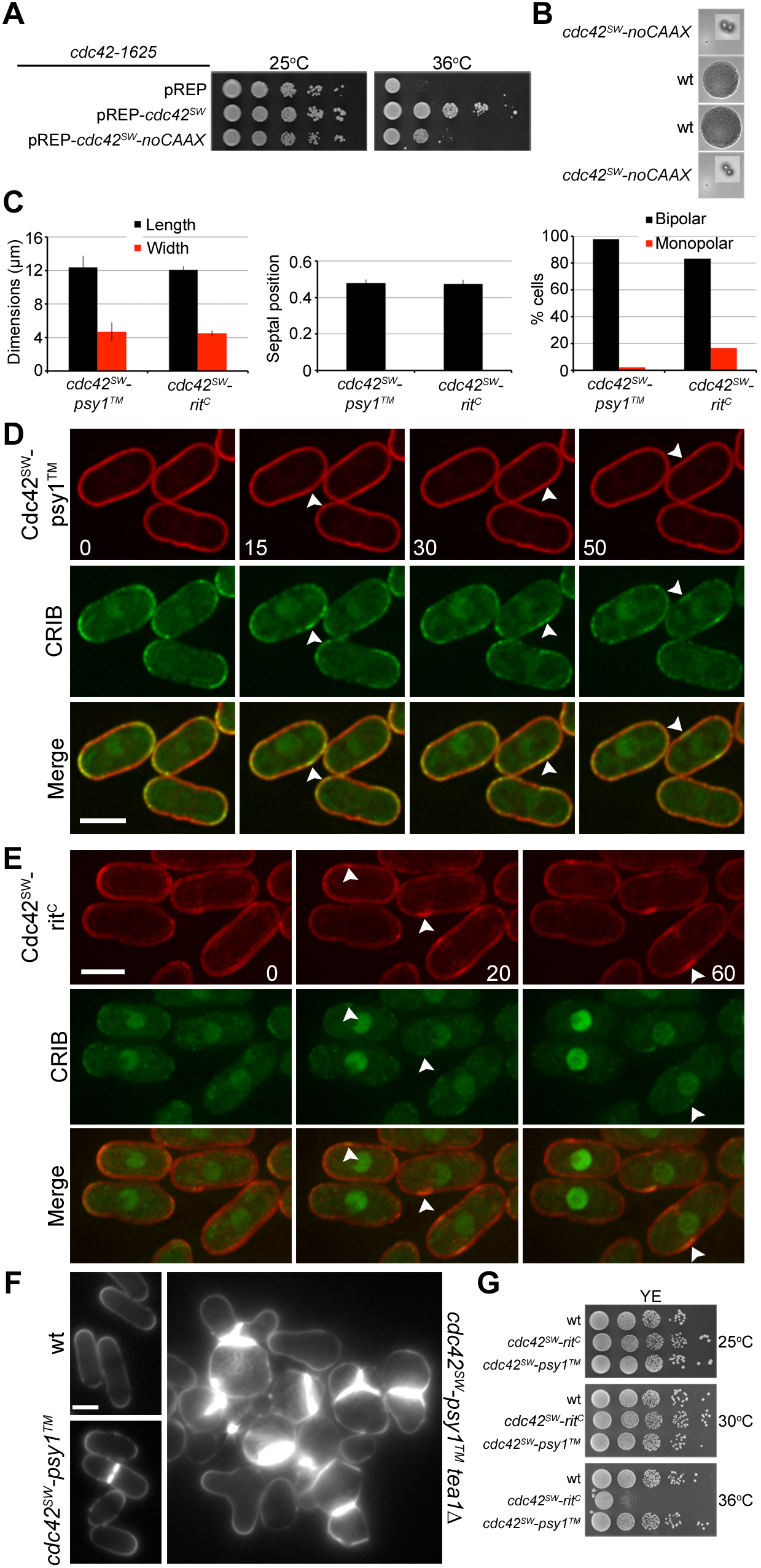

Supplement: S6 Fig — (A) Complementation of cdc42-1625 temperature sensitivity by pREP41-based plasmids. (B) Representative images of haploid cells from wt/cdc42-mCherry SW noCAAX:kanMX diploid tetrad dissection. Inset in top and bottom panels show high magnification. Note cdc42-mCherry SW noCAAX:kanMX cells arrest growth following first division attempt, likely because of zygotic wt Cdc42 perdurance in the first cell cycle. (C) Average cell length and width (left), septal position (middle) and percent cells growing in a mono- or bipolar manner (right) for cdc42 SW -psy1 TM and cdc42 SW -rit C strains (compare to S1D Fig). (D and E) Time-lapse images of Cdc42-mCherrySW–psy1TM (D) or Cdc42-mCherrySW–ritC (E) and CRIB-3GFP localization following treatment with LatA. Time is shown in minutes. T = 0 represents the first taken image, about 5 min after LatA addition. Arrowheads show examples of cell side accumulation of CRIB-3GFP and Cdc42-mCherrySW. (F) Calcofluor images of indicated strains in log phase. (G) 10-fold serial dilutions of indicated strains on YE. Bars = 5 μm. (TIF) [file pbio.1002097.s007.tif]

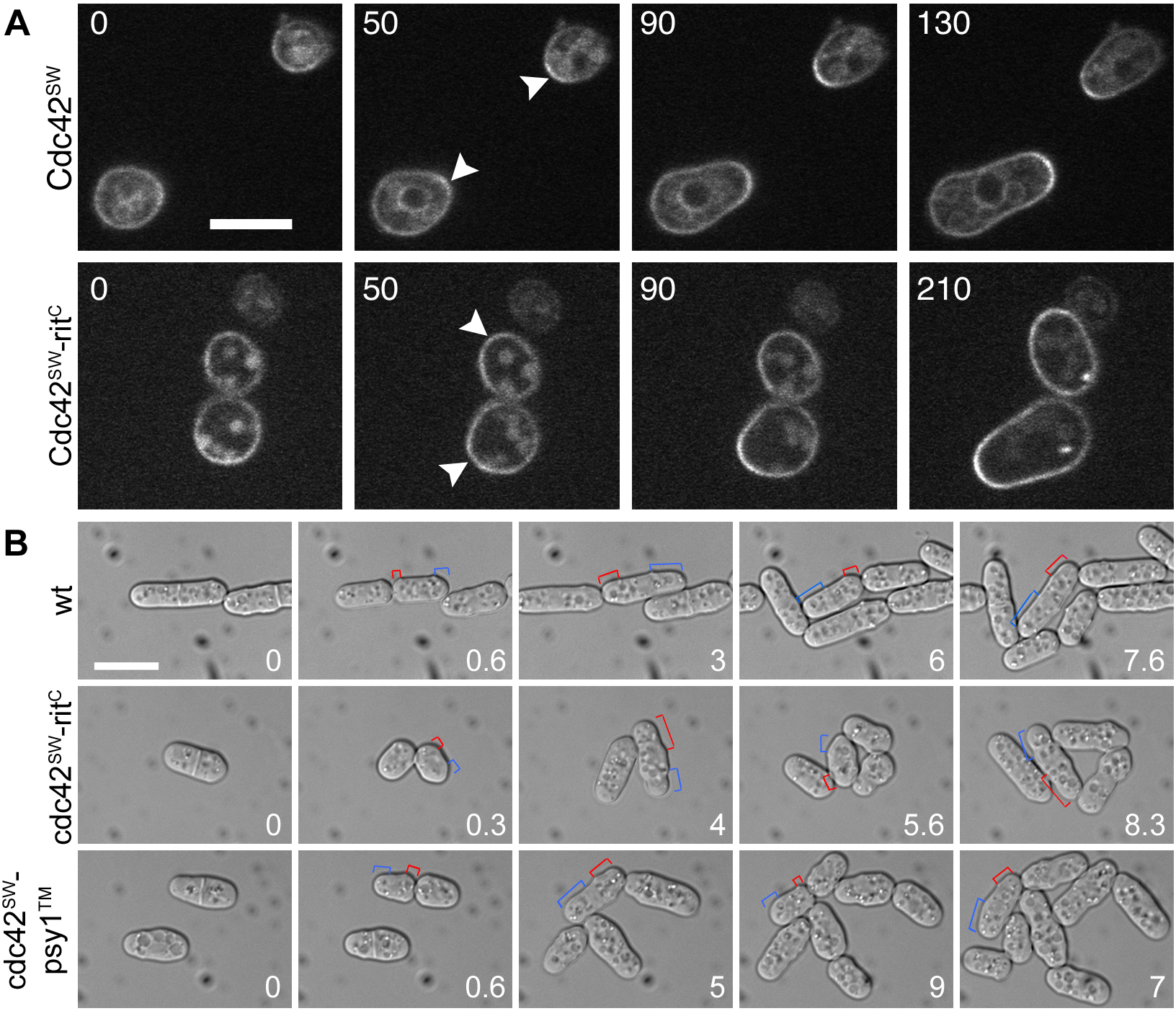

Supplement: S7 Fig — (A) Time lapse images of Cdc42-mCherrySW and Cdc42-mCherrySW-ritC fluorescence during spore outgrowth on YE. Arrowheads indicate site of fusion protein accumulation followed by polarized growth. Time is shown in minutes. Bar = 5 μm. (B) DIC images of indicated strains grown in microfluidic chambers with EMM. Red and blue lines represent cell growth at the old and the new cell end, respectively. Time is shown in h. Bar = 10 μm. (TIF) [file pbio.1002097.s008.tif]
