# Supplementary material for: Spontaneous Cdc42 Polarization Independent of GDI-Mediated Extraction and Actin-Based Trafficking
Source: PLoS Biol. 2015 Apr 2;13(4):e1002097. doi: 10.1371/journal.pbio.1002097 (PMC4383620; doi:10.1371/journal.pbio.1002097)
Supplement: S1 Table — (DOCX) [file pbio.1002097.s009.docx]

**Table S1. Fission and budding yeast strains used in this study**

***Schizosaccharomyces pombe***

| YSM995 | *h-* | Lab strain |
| --- | --- | --- |
| YSM2441 | *h+ cdc42-1625-kanMX leu1-32 ura4-D18 [pREP41]* | This work |
| YSM2442 | *h+ cdc42-1625-kanMX leu1-32 ura4-D18 [pREP41-cdc42]* | This work |
| YSM2443 | *h+ cdc42-1625-kanMX leu1-32 ura4-D18 [pREP41-cdc42-linker]* | This work |
| YSM2444 | *h+ cdc42-1625-kanMX leu1-32 ura4-D18 [pREP41-cdc42-GFP^SW^]* | This work |
| YSM2445 | *h+ cdc42-1625-kanMX leu1-32 ura4-D18 [pREP41-cdc42-mCherry^SW^]* | This work |
| YSM2446 | *h- cdc42-mCherry^SW^-kanMX* | This work |
| YSM2447 | *h90 cdc42-sfGFP^SW^-kanMX* | This work |
| YSM2448 | *sec8::nmt81-sec8-ura4+ cdc42-mCherrySW-bleMX scd2-GFP-hphMX* | This work |
| YSM2449 | *h+ cdc42-mCherry^SW^-kanMX ura4-294::Pshk1-ScGIC2 CRIB-3GFP-ura4+* | This work |
| YSM2450 | *cdc42-mCherry^SW^-kanMX scd1::kanMXura4-294::Pshk1-ScGIC2 CRIB-3GFP-ura4+* | This work |
| YSM2451 | *cdc42-mCherry^SW^-kanMX scd2::natMX ura4-294::Pshk1-ScGIC2 CRIB-3GFP-ura4+* | This work |
| YSM2452 | *cdc42-mCherry^SW^-kanMX gef1::kanMX ura4-294::Pshk1-ScGIC2 CRIB-3GFP-ura4+* | This work |
| YSM2453 | *cdc42-mCherry^SW^-kanMX orb2-34 ura4-294::Pshk1-ScGIC2 CRIB-3GFP-ura4+* | This work |
| YSM2454 | *cdc42-mCherry^SW^-bleMX rdi1::kanMX ura4-294::Pshk1-ScGIC2 CRIB-3GFP-ura4* | This work |
| YSM2455 | *cdc42-mCherry^SW^-kanMX tea4::kanMX ura4-294::Pshk1-ScGIC2 CRIB-3GFP-ura4+* | This work |
| YSM2456 | *cdc42-mCherry^SW^-kanMX tea4::kanMX* | This work |
| YSM2457 | *cdc42-mCherry^SW^-kanMX orb2-34* | This work |
| YSM2458 | *cdc42-mCherry^SW^-kanMX gef1::kanMX* | This work |
| YSM2459 | *cdc42-mCherry^SW^-bleMX for3::kanMX* | This work |
| YSM2460 | *cdc42-mCherry^SW^-bleMX rdi1::kanMX* | This work |
| YSM2461 | *cdc42-mCherry^SW^-kanMX end4::ura4+* | This work |
| YSM2122 | *h90 ura4-294::Pshk1-ScGIC2 CRIB-GFP3-ura4* | [[1](#_ENREF_1)] |
| YSM2462 | *h90 rdi1::kanMX ura4-294::Pshk1-ScGIC2 CRIB-3GFP-ura4* | This work |
| YSM2463 | *h+ cdc42-1625-kanMX leu1-32 ura4-D18[pREP41-cdc42-mCherry^SW^-rit^C^]* | This work |
| YSM2464 | *h+ cdc42-1625-kanMX leu1-32 ura4-D18 [pREP41-cdc42-mCherry^SW^-psy1^TM^]* | This work |
| YSM2465 | *cdc42-mCherry^SW^-psy1^TM^-kanMX ura4-294::Pshk1-ScGIC2 CRIB-3GFP-ura4+* | This work |
| YSM2466 | *h- cdc42-mCherry^SW^-psy1^TM^-kanMX* | This work |
| YSM2467 | *h- ade6+-GFP-psy1 leu1-32 ura4-D18* | This work |
| YSM2468 | *cdc42-mCherry^SW^-rit^C^-kanMX leu1-32 ura4-D18 ura4-294::Pshk1-ScGIC2 CRIB-3GFP-ura4+* | This work |
| YSM2469 | *h+ cdc42-mCherry^SW^-rit^C^-kanMX ade6- leu1-32 ura4-D18* | This work |
| YSM2470 | *cdc42-mCherry^SW^-rit^C^-kanMX sec8::nmt81-sec8-ura4+* | This work |
| YSM2471 | *h- cdc42-mCherry^SW^-rit^C^-kanMX ade6- leu1-32 ura4-D18* | This work |
| YSM1180 | *h- ade6-M210 leu1-32 ura4-D18* | Lab strain |
| YSM330 | *h- tea1::ura4+* | Lab strain |
| YSM2472 | *cdc42-mCherry^SW^-rit^C^-kanMX tea1::kanMX leu1-32 ura4-D18* | This work |
| YSM2473 | *cdc42-mCherry^SW^-kanMX cdc10-v50* | This work |
| YSM2474 | *cdc42-mCherry^SW^-rit^C^-kanMX cdc10-v50* | This work |
| YSM2475 | *cdc42-mCherry^SW^psy1^TM^-kanMX cdc10-v50* | This work |
| YSM2476 | *rdi1::kanMX* | This work |
| YSM2477 | *h- cdc42-mCherry^SW^-kanMX scd2-GFP-hphMX* | This work |
| YSM2478 | *h- cdc42-mCherry^SW^-bleMX scd2-GFP-hphMX rdi1::kanMX* | This work |
| YSM2479 | *for3-4myc-kanMX leu1- ura4- [pREP81-GFP]* | This work |
| YSM2480 | *h- ade6-M210 leu1-32 ura4-D18 [pREP41-cdc42-mCherry^SW^-noCAAX]* | This work |
| YSM2481 | *h- ade6-M210 leu1-32 ura4-D18 [pREP41-GFP-CAAX]* | This work |
| YSM2482 | *h+ cdc42-1625-kanMX leu1-32 ura4-D18 [pREP41-cdc42-mCherry^SW^-noCAAX]* | This work |
| YSM2614 | *cdc42-sfGFP^SW^-kanMX [pREP41-cdc42-mCherry^SW^]* | This work |
| YSM2615 | *cdc42-sfGFP^SW^-kanMX [pREP41-cdc42-Q61L-mCherry^SW^]* | This work |
| YSM2616 | *kanMX leu1-32 ura4-D18* | Lab strain |
| YSM2296 | *GFP-A_8_-cdc42-KanMX leu1-32 ura4-D18* | [[2](#_ENREF_2)] |

***Saccharomyces cerevisiae***

| YSC2 | *MATα his3Δ1 leu2Δ lys2Δ ura3Δ* | Lab strain |
| --- | --- | --- |
| YSC11 | *cdc42-ritC-GFP-kanMX his3Δ leu2Δ ura3Δ* | This work |
| YSC13 | *MATα cdc42-rit^C^-kanMX his3Δ leu2Δ lys2Δ met15Δ ura3Δ* | This work |

**Supplementary references**

1. Bendezu FO, Martin SG (2013) Cdc42 Explores the Cell Periphery for Mate Selection in Fission Yeast. Curr Biol 23: 42-47.

2. Coll PM, Rincon SA, Izquierdo RA, Perez P (2007) Hob3p, the fission yeast ortholog of human BIN3, localizes Cdc42p to the division site and regulates cytokinesis. Embo J 26: 1865-1877.
